# Integer linear programming for contrasting state interventions in Boolean networks

**DOI:** 10.7717/peerj.20676

**Published:** 2026-03-06

**Authors:** Costas Bampos, Vasileios Megalooikonomou

**Affiliations:** Computer Engineering and Informatics Department, School of Engineering, University of Patras, Patras, Greece

**Keywords:** Integer linear programming, Minimal intervention strategies, Gene regulatory networks

## Abstract

Drug discovery is a highly complex and time-consuming endeavor, often hindered by issues related to efficacy and safety, resulting in frequent late-stage drug attrition. Conventional strategies that rely on tightly controlling compounds’ physicochemical properties have had limited success. One reason is that pathogenic cells (*e.g*., in heart arrhythmias or seizures) often utilize the same pathways as healthy ones. Additionally, cellular heterogeneity and circuit hijacking by cancerous cells (*e.g*., MAP kinase signaling) complicate selective targeting. To address these challenges, network-based approaches are gaining traction as alternatives to traditional reductionist models. In this study, we propose a scalable method rooted in integer linear programming (ILP) principles to identify minimal intervention strategies that selectively modulate common nodes in structurally similar Boolean networks. Unlike previous approaches such as minimal cut sets or elementary modes (EMs), which struggle with large networks due to computational limitations, our ILP-based method offers both efficiency and selectivity. EMs are used only *post hoc* to validate final solutions. We evaluate our approach across five case studies, demonstrating its ability to modulate target nodes in one network while preserving their state in others. The results suggest this framework could support therapeutic design strategies aimed at precision targeting with reduced off-target effects.

## Introduction

### Overview

The article is organized as follows: In “Introduction” we motivate modeling choices (ordinary differential equations (ODEs) *vs*. logical formalisms) and argue for Boolean logic in large biological networks. Section “Problem Definition and Formalization” states the selective-modulation task, notation, and the high-level specification. In the “Methods” section we provide the integer linear programming (ILP) decision variables, gate encodings, linearizations, and the explicit algorithm used to generate and verify candidate interventions. In “Results” we report five case studies (synthetic examples, networks, and Kyoto Encyclopedia of Genes and Genomes (KEGG) comparisons) and runtime/verification benchmarks. Finally, the “Discussion” interprets biological and translational implications and limitations, and “Conclusions” summarize contributions.

### Discovering control variables in biological networks

Rapid advances in the computational sciences and the close resemblance of biological networks to digital circuits have enabled biologists to study complex biological phenomena using logical modeling techniques. Various approaches have been used to analyze genome-scale models and discover control variables (Wang & Albert, 2011; Langmead & Jha, 2009; Jia & Barabási, 2013; Li, Yang & Chu, 2015; Lu et al., 2015b; Motter, 2015). In topology-based methods (Borenstein & Feldman, 2009; Handorf, Ebenhöh & Heinrich, 2005), gene networks are abstracted as graphs, and graph-theoretic algorithms are employed to infer metabolic pathways. The logical methods (Lu et al., 2014, 2015b) are based on definitions of Boolean sets of rules for identifying whether particular reactions can proceed.

Previous studies have leveraged topology-based methods or logical rules to abstract metabolic and signaling networks. For example, after abstracting a metabolic network into a Boolean model, Smart, Amaral & Ottino (2008) and Lemke et al. (2004) conducted studies on how enzyme deletion could affect this network. Tamura, Takemoto & Akutsu (2010) working concurrently on many metabolic networks, developed an integer linear programming algorithm to define the minimum set of reactions whose removal modifies a target compound in only one selected network, leaving all the remaining reactions intact. Li et al. (2009) formally considered reducing indirect off-target side effects of enzyme inhibition by ensuring minimal elimination of non-target compounds. Branch-and-bound algorithms and answer set programming techniques have been employed to dynamically explore search spaces (Garg et al., 2013; Sridhar et al., 2008; Kaminski et al., 2013), as have methods applicable to more expressive logical operators (Sedghamiz et al., 2019).

Among the modeling formalisms, Boolean networks provide a computationally efficient, interpretable, and scalable framework for studying gene regulatory networks (GRNs), especially when detailed kinetic parameters are unavailable. Despite their simplicity, Boolean models capture essential dynamics of various biological processes—including cell differentiation, apoptosis, and cancer progression—through logical combinations of on/off states. Probabilistic Boolean networks (PBNs) have been introduced to incorporate stochasticity, but their computational burden is high. Our method opts for deterministic Boolean modeling, translated into ILP constraints for tractable exploration of intervention strategies.

The current approach proposed builds upon earlier efforts in network-based intervention strategies, particularly the work of Lu et al. (2015b), which introduced an integer programming framework for identifying minimal perturbations across multiple metabolic networks modeled in Boolean logic. While both methodologies share the objective of determining parsimonious intervention sets that affect specific nodes within Boolean networks, their target goals diverge significantly. Lu et al. (2015b) focus on computing interventions that alter the state of a target node in one selected network while preserving that node’s original state across others, thereby minimizing off-target effects. In contrast, our proposed framework advances this objective by introducing discriminative intervention strategies that enforce opposing outcomes for the same target node in structurally similar networks—achieving repression in a pathological context while maintaining activation in a healthy counterpart. This shift from passive preservation to active differentiation represents a conceptual refinement, with direct implications for precision therapeutic design in heterogeneous biological systems. This selective intervention strategy is particularly relevant in contexts where therapeutic modulation is desired in only a subset of similar biological circuits, such as targeting pathological cell subpopulations while avoiding collateral disruption to healthy tissues.

A notable methodological distinction lies in the computational workflows employed by the two approaches. Lu et al. (2015b) rely heavily on the enumeration of elementary modes (EMs) as a foundational step, using them to represent minimal functional pathways through metabolic networks and subsequently applying optimization techniques to identify intervention sets. In contrast, our proposed method reverses this sequence by employing ILP as the primary computational engine. Boolean regulatory logic is directly translated into linear constraints, allowing the intervention space to be explored without prior enumeration of EMs. EM analysis is used only as a *post hoc* validation step to assess the biological plausibility of the computed solutions. This reversal of computational workflow enables significant scalability advantages, as EM enumeration is known to become intractable in large or highly interconnected networks. By circumventing the need to exhaustively enumerate all EMs during optimization, the ILP-based approach can accommodate larger and more complex biological systems with greater computational efficiency.

### Binary logic and network analysis

In the last few years, drug discovery research has gradually shifted focus from the study of individual molecules to the study of signaling pathways and their complex interactions (Guney et al., 2016; Cheng, Kovács & Barabási, 2019; Pershad, Guo & Altman, 2020). The adoption of this holistic approach, which regards pathways and their network more important than individual biomolecules, aims to finely tune drugs toward precision medicine; increase the statistical power of functional annotation of the involved molecules; reduce the attrition rates associated with the drug discovery process; and search for new indications for already approved or investigational drugs with known side effects. During the early phases of drug discovery, system-based approaches have the potential to unravel the active mechanisms of disease and decipher signaling signatures in a more informed and detailed manner (Ma’ayan et al., 2007). These pathogenic biological routes can then be targeted through more precise therapeutic interventions, with the potential to reverse those ill-regulated processes and their interactions. Finally, a single marker might not be sufficiently specific or sensitive enough to correctly identify pathogenic cells. However, combining multiple markers when targeting circuits could improve specificity (Anderson, Voigt & Arkin, 2007).

The two most distinguished classes of network analysis are as follows:

- **Topological analysis** involves extracting structural network properties such as scale-free, small-world, motif recognition, community detection or centrality measures.
- **Network identification** describes the signal transduction process between the network’s nodes.

The focus of the present article is the second class. Although one of the most efficient formalisms for modeling biological networks is ordinary differential equations (ODEs), their selection as a modeling approach is restricted mainly to small-sized interrogated signaling networks, especially due to excessive computational costs and the excessive amount of experimental data needed to describe the association-dissociation constants and the stoichiometry at hand. To address large-scale networks, a more efficient approach for structure identification would be logic-based modeling (Mitsos et al., 2012).

Although quantitative and continuous mechanistic models are preferred for system-based analysis, it has been shown that the emergent properties of biological systems may be largely established by complex interactions among network components (Kitano, 2002; Barabási & Oltvai, 2004), implying that the paucity of comprehensive kinetic parameters might not be the sole way of studying a network’s dynamic properties. Boolean network models are the simplest discrete mathematical models, and occupy only two states for each node in biological networks without other explicit dynamical rules (Kauffman, 1969).

Binary logic formalism has been widely used as a modeling tool for gene network analysis due to its many inherent advantages: all the nodes and their regulators in a Boolean network are linked in a simple, predefined way *via* logical operators; the computational complexity is low; and the results are easy to interpret (de Jong, 2002). Gene regulatory networks (GRNs) can be simulated with on/off dynamics even when the biochemical kinetic parameters of one or more biological processes are unknown or inaccurate; moreover, studying the effects of knocking out or overexpressing a gene is straightforward. They are also especially useful when the networks contain different species of biological entities, such as mRNAs, transcription factors and DNA (Lu et al., 2015a). Finally, the inherent properties of GRNs such as probabilistic distributions of expression levels, different cellular states or phenotypes, or various oscillatory processes of biological origin, can be easily captured (Lovrics et al., 2014). Boolean networks have been applied to many biological processes and have provided key insights for various pathophysiological systems such as the immune system (*e.g*., Ruiz-Cerdá et al., 2016; Saez-Rodríguez et al., 2007) and cancer (*e.g*., von der Heyde et al., 2014; Flobak et al., 2015; Lu et al., 2015a).

A Boolean network represents a series of interconnected *N*-vector elements ${\bf{x}}$, where each element can exist in one of two possible functional states: ${x_{i}} \in \{ 0,1\}$ for $i = 1, \ldots ,N$. The modeling of a Boolean network can be formulated as follows:

Suppose the network is represented by a directed graph *G(V, F)*, where $V = \{ {x_{1}}, \ldots ,{x_{N}}\}$ is the set of nodes representing the states of a regulatory system of *N* elements, and *F* is a set of Boolean functions. A Boolean function or rule ${f_{i}}({x_{{i_{1}}}}, \ldots ,{x_{{i_{k}}}})$ is assigned to each node ${x_{i}}$, where *k* denotes the in-degree of the node. The function ${f_{i}}$ determines the state of node ${x_{i}}$ at time $t + 1$ based on the states of its *k* regulators at the previous time step *t*, as follows:

(1) $${x_{i}}(t + 1) = {f_{i}}\left( {{x_{{i_{1}}}}(t),{x_{{i_{2}}}}(t), \ldots ,{x_{{i_{k}}}}(t)} \right).$$

Due to the stochastic aspects of gene expression, the above formalisms could be extended to explicitly address the dynamics of regulatory systems. To overcome the inherent deterministic nature of the original Boolean models, Shmulevich et al. (2003) developed probabilistic Boolean networks (PBNs) for gene networks that are robust under uncertainty. The xi nodes are assigned a vector of possible Boolean functions l(i), i = (1,…, N), which predicts the value of a node xi at the next time point. Due to the high computational cost of the probabilistic model, however, we implemented the simpler approach.

**Figure 1:**
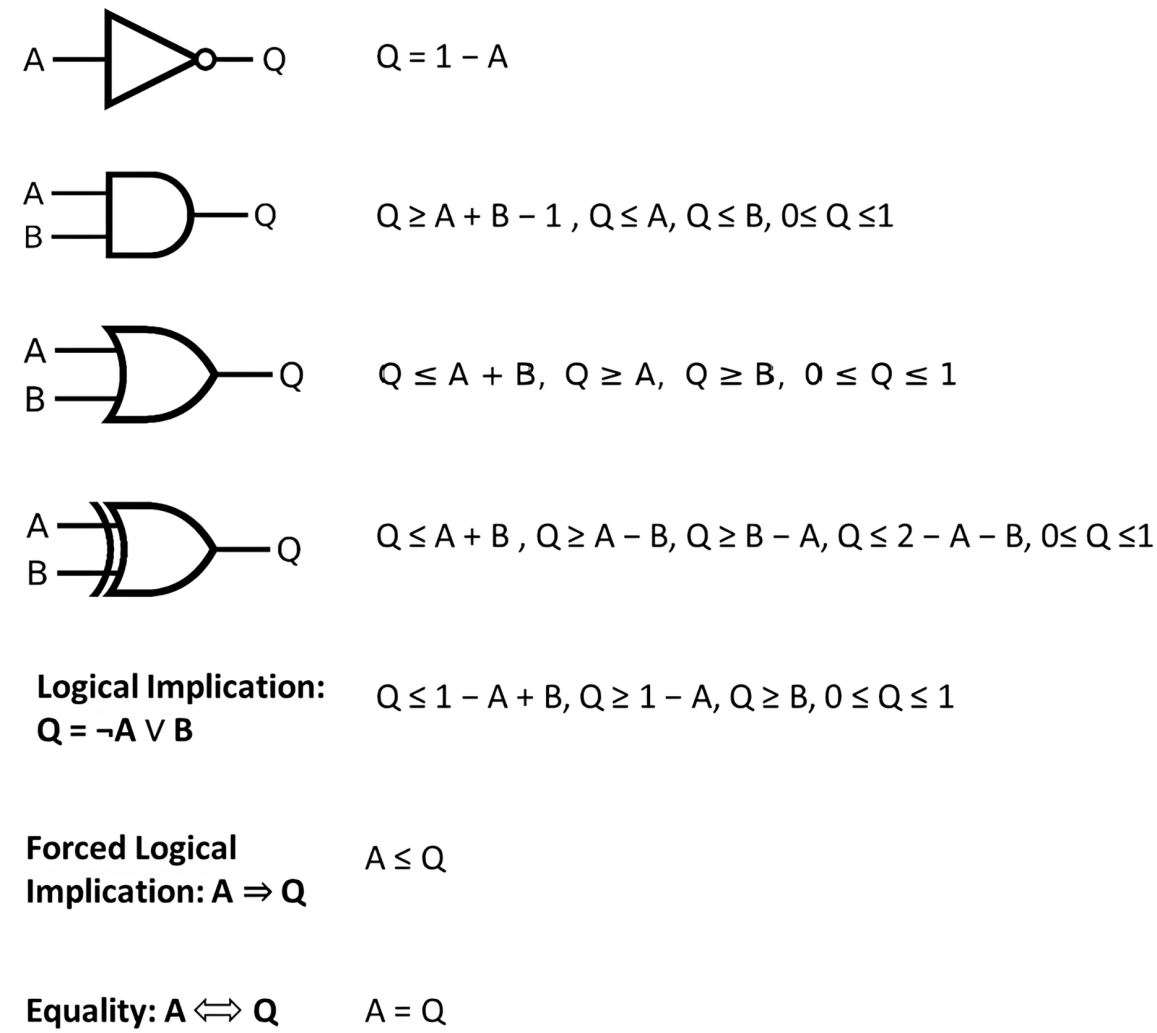
Converting binary operators to linear constraints.

To optimally explore the solution space of the Boolean model, we translated the original problem into an integer programming formalization by representing every Boolean constraint *via* linear equations or inequalities (Fig. 1):

Note that the binary-operator encodings illustrated in Fig. 1 are further presented algebraically as Eq. (6)

### Biological networks in cancer

Cancer cells often hijack core signaling motifs *via* mutations or rewiring, creating an opportunity to target aberrant subpopulations without disrupting healthy tissue.

In normal biological networks, intracellular and cell–cell communications are transmitted *via* networked molecules, which relay messages through protein-mediated interactions. The interaction specificity is further enhanced by post-translational modifications, which confer spatiotemporal regulation of the network components. Among these regulatory mechanisms, the tyrosine phosphorylation signaling pathway has received considerable attention due to its importance in interacting with hallmark processes such as differentiation, apoptosis, proliferation and motility, as well as in drug response and cancer remission (McGee et al., 2017). The human genome, which harbors 90 protein tyrosine kinases and more than 10,000 tyrosine phosphorylation sites (Liu, Engelmann & Nash, 2012; Tan et al., 2009), comprises an enormous, intricate pTyr network. However, it is also well known that all the genes that encode tyrosine kinases and the majority of their SH2-containing substrates have been identified as cancer-driving genes (Forbes et al., 2011).

Although there are many safety mechanisms in place to ensure robust function and avert possible misfiring of the pTyr signaling network, cancerous cells and some pathogenic bacteria are notoriously known for hijacking signaling system components and motifs in their host through gene mutations (Li et al., 2012) or by injecting a virulence factor during infection (Gruenheid et al., 2001; Backert, Tegtmeyer & Selbach, 2010). In recent years, the low cost and escalation of advancements in whole-genome and whole-exome sequencing technology from tumor samples have led to the construction of massive genome-scale models. The identified perturbed genes are widely accepted to be organized and act within abnormally wired networks that are causal drivers of tumor progression (Carter et al., 2009).

Comparative network-based analysis in combination with modeling tools has been used to analyze raw proteomics data from various genes and their isoforms in wild-type and diseased states. After generating a protein interaction network for each isoform complex, extensive rewiring of the resulting interaction networks becomes apparent, and new functional ontologies can be defined. In many of the annotated pathological communities, hub nodes are associated with aberrant pTyr signaling, which drives many of the fundamental biological processes that accompany tumor initiation and progression (Paliouras et al., 2011). Mutated cancer driver genes have been shown to be enriched in positive network motifs (Cui et al., 2006), whereas the properties of frequently occurring network regulatory motifs lead to functional reorganization of these signaling modules and network rewiring (Paliouras et al., 2011).

The type and frequency of mutated genes in patients differ not only between cancer types but also within the same cancerous tissue. Several causes contribute to intratumor heterogeneity that may affect key signaling pathways (Sun & Yu, 2015). The minority of cancer cell populations may have an impact on the progression of the disease more than the bulk of the cells in a tumor (Swanton, 2012). Since there could be a tumorigenic hierarchy of cells, an adaptive therapeutic approach in which critical clones within solid tumors are controlled but not eliminated has been proposed to contain the progression of the disease (Gatenby et al., 2009).

Although this rewiring of intracellular compounds plays an important role in tumorigenesis, it also presents an opportunity to electively target and modify only the pathogenic cell population while leaving normal cells intact.

### Biological networks in other cell types

The same concept is not explicit to cancerous cells but extends to other domains, including neural circuits (*e.g*., epilepsy, depression) and microbial communities, where selective modulation could be therapeutically beneficial.

Despite the fact that the above examples describe the pathology of cancer, there are many more cases where both abnormal and normal rewiring of evolutionary origins can be mustered towards therapy. Many diverse populations of neurons in the mammalian brain have been discovered that are functionally incorporated into different circuits with different roles, even in very small nuclei such as dopaminergic neurons in the substantia nigra pars compacta (SNc) (Lerner et al., 2015). Targeting of SNc cell types could be therapeutic for diseases such as depression, addiction, schizophrenia, epilepsy and movement disorders. Epilepsy is a disease in which four different circuits coexist (an ictal that causes epileptic seizures, an interictal circuit that sustains the epileptic focus between seizures, a preictal circuit that initiates epileptic seizures, and normal brain tissue). There is evidence that in some prevalent types of neocortical epilepsies, cell activity is associated with kinase phosphorylation (Curia et al., 2013; Rakhade et al., 2005). Finally, various coexisting bacteria populations, either in industrial fermentation (Hatzimanikatis, Floudas & Bailey, 1996a; Hatzimanikatis, Floudas & Bailey, 1996b) or in the human intestine (Monteiro et al., 2019), must be kept in proper balance, either to maximize the production of a material or for good health.

## Materials and Methods

### Problem definition and formalization

Objective: Given two or more networks with slightly different node connectivity, identify the minimal set of nodes whose activation or repression across all networks will modulate a specific target node in one designated network, while leaving the corresponding target nodes in the remaining networks unaffected.

Let ${G_{A}} = ({V_{A}},{E_{A}})$ and ${G_{B}} = ({V_{B}},{E_{B}})$ be directed graphs representing Boolean regulatory networks *A* and *B*, respectively. Each node ${x_{i}} \in V$ denotes a biological element (*e.g*., a gene or protein) and takes a binary state ${x_{i}} \in \{ 0,1\}$. An edge $({x_{j}} \to {x_{i}}) \in E$ indicates that node ${x_{j}}$ regulates ${x_{i}}$. The state of each node is updated according to a Boolean function:

(2) $${x_{i}}(t + 1) = {f_{i}}({x_{{i_{1}}}}(t),{x_{{i_{2}}}}(t), \ldots ,{x_{{i_{k}}}}(t)),$$

where ${f_{i}}:{\{ 0,1\} ^k} \to \{ 0,1\}$, and $\{ {x_{{i_{1}}}}, \ldots ,{x_{{i_{k}}}}\}$ are the parent nodes of ${x_{i}}$.

**Notation.** We adopt the following conventions to avoid ambiguity. Boldface vectors denote full network states, ordinary letters denote single nodes. Thus ${\bf{x}} = ({x_{1}}, \ldots ,{x_{N}})$ is a network state vector and ${x_{i}}$ the $i$-th node. We reserve ${x^*}$ for the *target node* (a single node index), and we write network-specific node values using a network superscript: $x_{A}^*$ and $x_{B}^*$ denote the value of the target node in networks *A* and *B*, respectively. Desired values are denoted ${d_{A}},{d_{B}} \in \{ 0,1\}$ and we require ${d_{A}} \ne {d_{B}}$ for the selective modulation task. When we write ${{\scr S}_A}(x_{A}^* = d)$ we mean the family of node-sets (interventions) that, when applied to network *A*, ensure that the post-intervention value of the node indexed by ${x^*}$ equals $d$.

We define:

-
${{\scr S}_A}(x_{A}^* = d)$: the set of interventions in *A* that result in $x_{A}^* = d$,
-
${{\scr S}_B}(x_{B}^* = d^\prime )$: the set of interventions in *B* that result in $x_{B}^* = d^\prime$.

We seek an intervention set *S* that yields opposite target values in the two networks, *i.e*., the same intervention must force the target node to one value in network *A* and to the other value in network *B*:

(3) $$S \in {{\scr S}_A}(x_{A}^* = {d_{A}})\quad \rm {and} \quad S \in {{\scr S}_B}(x_{B}^* = {d_{B}}),$$

where ${d_{A}},{d_{B}} \in \{ 0,1\}$ and ${d_{A}} \ne {d_{B}}$. Equivalently, the ILP must produce a vector *u* (and reconstructed set $S = \{ i:{u_{i}} = 1\}$) such that the post-intervention target values satisfy $x_{A}^* = {d_{A}}$ and $x_{B}^* = {d_{B}}$.

### ILP formulation and constraints

Boolean functions are translated into linear constraints to enable ILP. The following transformations are used:

#### ILP decision variables and linear encoding

We list the ILP decision variables used in our implementation and provide the linear constraints used to implement the linkage between perturbations and node values.

**ILP decision variables (per network).**

-
$x_{i}^A,x_{i}^B \in \{ 0,1\}$: final binary value of node *i* in networks *A* and *B* after any perturbation.
-
$\hat x_{i}^A,\hat x_{i}^B \in \{ 0,1\}$: value computed by the unperturbed Boolean logic (auxiliary).
-
${u_{i}} \in \{ 0,1\}$: perturbation flag; ${u_{i}} = 1$ iff node *i* is directly perturbed (*i.e*., $i \in S$).
-
$z_{i}^A,z_{i}^B \in \{ 0,1\}$: enforced value for node *i* under perturbation (allows network-specific enforced states).
- Additional auxiliary gate variables *y* are introduced to represent intermediate gate outputs where needed.

**Boolean gate encoding.** Each logical relation is implemented *via* standard linear inequalities using an auxiliary binary for the gate output:

(4) $$\eqalign{ {\mathrm{AND: }}\;w & = {\mathop \wedge \limits _{i = 1}^k}{a_i}\quad \Longrightarrow \quad w \le {a_i}\;\;(\forall i = 1, \ldots ,k),\;w \ge \sum\limits_{i = 1}^k {{a_i}} - (k - 1), \\ {\mathrm{OR: }}\;w & = {\mathop \vee _{i = 1}^k}{a_i}\quad \Longrightarrow \quad w \ge {a_i}\;\;(\forall i = 1, \ldots ,k),\;w \le \sum\limits_{i = 1}^k {{a_i}} , \\ {\mathrm{NOT: }}\;w & = \neg a\quad \Longrightarrow \quad w = 1 - a. \cr }$$

**Linearization of the perturbation linkage.** The nonlinear equality

${x_{i}} = (1 - {u_{i}}) \cdot {\hat x_{i}} + {u_{i}} \cdot {z_{i}}$

is implemented for binary variables by the following *four* linear constraints (equivalent for ${u_{i}},{\hat x_{i}},{z_{i}} \in \{ 0,1\}$):

(5) $$\matrix{ {{x_{i}} - {{\hat x}_i} \le {u_{i}},} \cr {{{\hat x}_i} - {x_{i}} \le {u_{i}},} \cr {{x_{i}} - {z_{i}} \le 1 - {u_{i}},} \cr {{z_{i}} - {x_{i}} \le 1 - {u_{i}}.} \cr }$$

When ${u_{i}} = 0$ these force ${x_{i}} = {\hat x_{i}}$, and when ${u_{i}} = 1$ they force ${x_{i}} = {z_{i}}$.

**Practical note on notation and implementation.** The set-based statement Eq. (3) is a high-level specification: in the ILP we do not use a set-valued decision variable *S*. Instead, interventions are represented by binary decision variables ${u_{i}}$ and the intervention set is reconstructed after solving as

$$S = \{ i:{u_{i}} = 1\} .$$

Consequently, enforcing Eq. (3) in the MILP is achieved by (i) encoding the unperturbed logic to compute auxiliary variables ${\hat x^A},{\hat x^B}$, (ii) linking $\hat x,z$ and $u$ *via* linearization Eq. (5), and (iii) adding the explicit target constraints $x_{A}^* = {d_{A}}$ and $x_{B}^* = {d_{B}}$ (with ${d_{A}} \ne {d_{B}}$). This paragraph clarifies the mapping between the set notation and the concrete ILP variables used in the implementation.

### How equations are implemented in ILP

For clarity, the set-membership statements and equalities in the text are implemented as follows in our MILP:

- The condition $S \in {{\scr S}_A}(x_{A}^* = d)$ is enforced by (i) adding the logical/auxiliary constraints that compute the unperturbed outputs ${\hat x^A}$, (ii) introducing ${u_{i}}$ and $z_{i}^A$ and the linearization Eq. (5), and (iii) constraining $x_{A}^* = d$. The solver then selects ${u_{i}}$ and $z_{i}^A$ so that the resulting ${x^A}$ satisfy the equality.
- *S* is not a primitive ILP decision variable; it is reconstructed from the ${u_{i}}$ values after solve (see previous paragraph).
- Any textual constraint of the form “ $x_{i}^A = x_{i}^B$ for all $i \;\notin \;S$” is enforced by the combination of the four linear constraints (5) for both networks (so that when ${u_{i}} = 0$ the two networks both use their unperturbed $\hat x$ values) together with an explicit equality $x_{i}^A-x_{i}^B = 0$ added conditionally when ${u_{i}} = 0$. Practically, this conditional equality is realized by the linear implications above combined with the model construction described (*i.e*., the solver will set ${u_{i}}$ to 1 for nodes where the networks must differ).

**Remark.** Equations (4) and (6) give equivalent linear encodings of Boolean gates. Equation (4) states the general k-ary formulas, whereas Eq. (6) presents the common binary (two-input) special case used elsewhere in the manuscript.

(6) $$\matrix{ {{\mathrm{AND:\;}} z \le x,\;z \le y,\;z \ge x + y - 1,}\hfill \cr {{\mathrm{OR:\;}} z \ge x,\;z \ge y,\;z \le x + y,}\hfill \cr {{\mathrm{NOT:\;}} z = 1-x.}\hfill \cr }$$

To model perturbations, we introduce binary variables ${u_{i}} \in \{ 0,1\}$, where ${u_{i}} = 1$ indicates that node *i* is perturbed. The target node constraints are expressed as:

(7) $$x_{A}^* = d\quad \rm {and} \quad x_{B}^* = d^{\prime} ,$$

where $d \ne d^{\prime}$.

To ensure that only perturbed nodes differ across networks, we enforce the constraint:

(8) $$x_{i}^A = x_{i}^B\quad \forall \;i \;\notin \;S.$$

To ensure selectivity of the intervention we require that the same intervention set *S* forces opposite target values in the two networks Eq. (3).

**Objective Function** The goal is to minimize the number of perturbed nodes:

(9) $$\min \sum\limits_{i} {{u_{i}}} .$$

**Perturbation Linkage Constraint.** We link node values to perturbation status:

(10) $${x_{i}} = {\hat x_{i}}\, (1 - {u_{i}}) + {z_{i}}\, {u_{i}},$$

where ${\hat x_{i}}$ is the value determined by the unperturbed Boolean logic, and ${z_{i}} \in \{ 0,1\}$ is the enforced state under perturbation.

**Post-Validation *via* Elementary Modes.** An *elementary mode (EM)* is defined on a stoichiometric matrix $N \in {\mathbb R}{^{m \times r}}$ (rows = species, columns = reactions) as a nonzero flux vector $v \in {\mathbb R}^{r}$ that (i) satisfies the steady-state constraint $Nv = 0$, (ii) respects reaction directionality (for irreversible reactions ${v_{j}} \ge 0$; reversible reactions may be represented by splitting ${v_{j}} = v_{j}^ + - v_{j}^ -$ with $v_{j}^ \pm \ge 0$), and (iii) is *support-minimal*: there exists no nonzero $w \ge 0$ with $Nw = 0$ and $\operatorname {supp}(w)\; \subsetneq \; \operatorname {supp}(v)$. In words, an EM is a steady-state flux that cannot be realized by a strictly smaller set of reactions.

To provide a reproducible, checkable minimality test we use the following MILP formulations. Let binary variables ${y_{j}} \in \{ 0,1\}$ indicate whether reaction *j* is active and continuous fluxes ${v_{j}} \ge 0$ (or split fluxes for reversible reactions) represent reaction rates. Enforce steady state and big-*M* linking:

$Nv = 0,\qquad 0 \le {v_{j}} \le M\, {y_{j}}\quad \forall j,$

and require a nonzero flux by $\sum\nolimits_{j} {{v_{j}}} \ge {\varepsilon _{{{\mathrm{tot}}}}}$ (with ${\varepsilon _{{{\mathrm{tot}}}}} > 0$ small). Then:

- **Existence of a steady flux supported on a candidate set *S*.** Fix ${y_{j}} = 1$ for $j \in S$ and ${y_{j}} = 0$ for $j\;\notin\; S$; test feasibility of the constraints above. Feasibility certifies that a steady flux with support contained in *S* exists (but not minimality).
- **Support-minimality (EM validation).** Solve the MILP $\min \sum\nolimits_{j} {{y_{j}}}$ subject to $Nv = 0$, $0 \le {v_{j}} \le M{y_{j}}$, $\sum\nolimits_{j} {{v_{j}}} \ge {\varepsilon _{{{\mathrm{tot}}}}}$, and $\, {y_{j}} = 0$ for $j\; \notin\; S$. If the optimal objective value equals |*S*| then no strictly smaller-support steady flux exists inside *S* and *S* is support-minimal (hence an EM); if the optimum is < |*S*| then *S* is not minimal.

Choose *M* larger than any physically/plausibly attainable flux (or use a computed upper bound) and ${\varepsilon _{{{\mathrm{tot}}}}}$ small but numerically distinguishable from zero.

When applicable, EM validation is used as a *biological plausibility* check: we prefer ILP solutions whose perturbed-node support corresponds to an EM in network *A* but not in network *B*, *i.e*.,

(11) $$S \in {\mathrm{EM}}_{A}\quad {\rm {and}} \quad S\; \notin \; {\mathrm{EM}}_B.$$

This ensures that the selected intervention corresponds to a minimal steady-state pathway in the stoichiometric sense in network *A* while being infeasible or non-minimal in *B*. All of the conditions are implemented in four different problems formulated as ILPs, specifically designed to test the validity of the proposed algorithm:

- **Combinatorial Node Modulation in one network.**
- **Selective repression of a node included in both networks when outdegree of the target node $X = 0$.**
- **Selective repression of a node included in both networks when outdegree of the target node $X > 0$.**
- **Selective repression of a node included explicitly in one network.**

As a final test, we implement our model to two slightly different, medium-sized metabolic networks. All the schematics and the reactions were downloaded from Kyoto Encyclopedia of Genes and Genomes (KEGG) (Kanehisa & Goto, 2000), through PathView (Luo & Brouwer, 2013), in png and xml files, respectively. To list the reactions of the two networks along with their differences, we used KEGGtranslator (Wrzodek, Dräger & Zell, 2011) to convert xml to sbml files, and then process the final file through CellNetAnalyzer (von Kamp et al., 2017).

The key steps of our network motif detection approach are summarized in Algorithm 1.

**Algorithm 1:** Minimal selective intervention in boolean networks (explicit ILP construction and verification).

** Input:** Two Boolean networks *A* and *B* (logic tables/gate descriptions), common node set ${V_{A}} \cap {V_{B}}$, target node index ${x^*}$, desired target values ${d_{A}},{d_{B}} \in \{ 0,1\}$ with ${d_{A}} \ne {d_{B}}$.
** Output:** Minimal intervention set(s) $S \subseteq {V_{A}} \cap {V_{B}}$ such that post-intervention $x_{A}^* = {d_{A}}$ and $x_{B}^* = {d_{B}}$.
** ILP decision variables (one set per node *i* and per network where indicated):**
• $x_{i}^A,x_{i}^B \in \{ 0,1\}$: final value of node *i* in networks *A* and *B* after interventions.
• $\hat x_{i}^A,\hat x_{i}^B \in \{ 0,1\}$: unperturbed value computed by the Boolean logic (auxiliary variables).
• ${u_{i}} \in \{ 0,1\}$: perturbation flag; ${u_{i}} = 1 \Leftrightarrow i \in S$.
• $z_{i}^A,z_{i}^B \in \{ 0,1\}$: enforced value for node *i* when perturbed (allows network-specific forced values).
• Auxiliary gate variables ${g_{k}} \in \{ 0,1\}$ as needed to represent intermediate gate outputs when a node’s logic is decomposed into gates.
** (1) Encode the Boolean logic.**
For each node *i* in network *A* express its Boolean function $f_{i}^A$ as a logic circuit using AND, OR, and NOT gates (introducing auxiliary gate variables for intermediate outputs). Then add the corresponding linear inequalities for each gate:
** k-ary AND** $w={\wedge}^{k}_{j=1}a_{j}:$
$\left\{ {\matrix{ {w \le {a_{j}}} \hfill & {\rm {for\;}} = 1, \ldots ,k, \hfill \cr {w \ge \sum\nolimits_{j = 1}^k {{a_{j}}} - (k - 1).} \hfill } } \right.$
** k-ary OR** $w = \bigvee\nolimits^{k}_{j=1} a_j$:
$\left\{ {\matrix{ {w \le {a_{j}}} \hfill & { {\rm for\;}{j} = 1, \ldots ,k,} \hfill \cr {w \ge \sum\nolimits_{j = 1}^k {{a_{j}}} .} \hfill } } \right.$
** NOT** $w = \neg a$: $w = 1\mathrm{-}a$.
Use these gate constraints to enforce $\hat x_{i}^A$ (the unperturbed output) for every node *i*. Repeat the same construction for network *B* to enforce $\hat x_{i}^B$.
** (2) Link perturbation flags to final node values (exact linearization).**
For each node *i* and for each network (replace ^⋅^ by ^A^ or ^B^), enforce the algebraic perturbation linkage:
(12) $$x_{i}^ \cdot \; = \;(1 - {u_{i}})\, \hat x_{i}^ \cdot + {u_{i}}\, z_{i}^ \cdot .$$
This equality is implemented exactly for binary variables by the following four linear inequalities:
(13) $$\matrix{ {x_{i}^ \cdot - \hat x_{i}^ \cdot \le {u_{i}},} \hfill \cr {\hat x_{i}^ \cdot - x_{i}^ \cdot \le {u_{i}},} \hfill \cr {x_{i}^ \cdot - z_{i}^ \cdot \le 1 - {u_{i}},} \hfill \cr {z_{i}^ \cdot - x_{i}^ \cdot \le 1 - {u_{i}}.} \hfill \cr }$$
These force $x_{i}^ \cdot = \hat x_{i}^ \cdot$ when ${u_{i}} = 0$ and $x_{i}^ \cdot = z_{i}^ \cdot$ when ${u_{i}} = 1$.
** (3) Ensure shared nodes remain equal when not perturbed.**
To guarantee that a shared node *i* has identical final values in both networks when it is *not* perturbed ( ${u_{i}} = 0$), add the two linear inequalities:
(14) $$\matrix{ {x_{i}^A-x_{i}^B \le {u_{i}},} \cr {x_{i}^B-x_{i}^A \le {u_{i}}.} \cr }$$
When ${u_{i}} = 0$ both inequalities reduce to $x_{i}^A = x_{i}^B$; when ${u_{i}} = 1$ they place no restriction beyond the bounds $x_{i}^ \cdot \in \{ 0,1\}$.
** (4) Target constraints and objective.** Add the two target constraints:
$x_{A}^* = {d_{A}},\quad x_{B}^* = {d_{B}},$
and the objective to minimize the number of perturbed nodes:
$\min \;\sum\nolimits_{i} {{u_{i}}} .$
** (5) Solver usage and solution pool.** Use the MILP solver’s solution-pool/enumeration features (or repeated solves with symmetry-breaking) to collect all optimal supports (all solutions with minimal objective). Each solution provides a vector **u** and therefore a candidate support $S = \{ i:{u_{i}} = 1\}$.
** (6) Verify minimality and EM membership.** For each candidate support *S* extracted from the MILP solutions perform the following checks:
• **If stoichiometric/EM data are available:** verify whether *S* corresponds to an EM in network *A* and not in network *B*. Concretely, enumerate EMs of the stoichiometric model and check set inclusion:
$\hskip40pt S \in {\rm EM_{A}}\quad{\rm and}\quad S \in {\rm EM_{B}}.$
Only candidates passing this check are accepted as biologically minimal steady-state interventions.
• **If EMs are not defined (pure Boolean networks):** perform the MILP minimality test: fix all *u*_*i*_ for $i \in S$ to 1, and for each $j \in S$ attempt to re-solve with ${u_{j}} = 0$; if any such re-solve is feasible then *S* is not minimal and is discarded. If no single-element removal yields feasibility then *S* is minimal with respect to single-element removals (the test can be extended to larger subsets if stricter minimality is required).
** (7) Return.** Return all candidate supports *S* that (a) satisfy the target constraints, (b) were produced as optimal MILP solutions, and (c) pass the chosen minimality/EM checks.

## Results

### Combinatorial node modulation in a single network

We first examine the simplest case: identifying the minimum set of nodes that must be perturbed to inhibit a single target node in one network. We explicitly treat the single-network case as the simplest instantiation of our ILP framework: in that run we construct the MILP only for the single Boolean network (declaring ${x_{i}},{\hat x_{i}},{u_{i}},{z_{i}}$ and any gate auxiliaries for that network), enforce the single-network target constraint $x_{A}^* = d$, omit all inter-network equality/selectivity constraints (*i.e*. the $x_{i}^A = x_{i}^B$ implications and the ${d_{A}} \ne {d_{B}}$ requirement), and minimize $\sum\nolimits_{i} {{u_{i}}}$.

**Figure 2:**
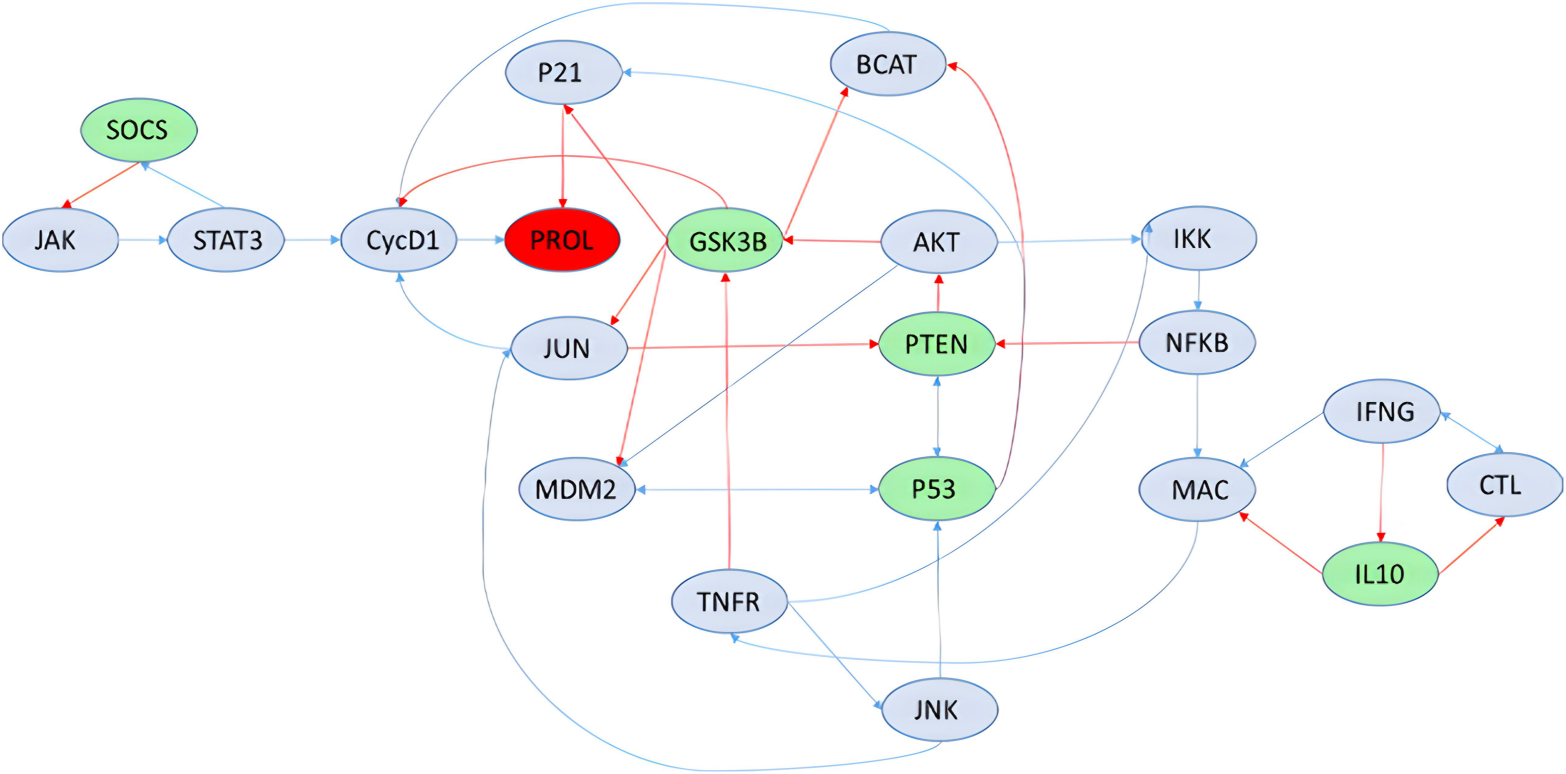
The reduced colitis-associated colon cancer network.

Using the reduced colitis-associated colon cancer (CACC) Boolean network (21 nodes, from Choo, Park & Cho (2019)), we target the deactivation of the node PROL. Our ILP-based model identifies the required interventions: for the PROL node to be deactivated, green nodes must be assigned to 1 and blue nodes to 0, that is, to inhibit node PROL, we must activate nodes SOCS, GSK3B, PTEN, P53, IL10 (Fig. 2). These results are consistent with previous findings and confirm the model’s validity in capturing known therapeutic targets Eq. (11).

### Selective repression of a node included in both networks ( ${{\mathrm{outdegree}}} = 0$)

**Figure 3:**
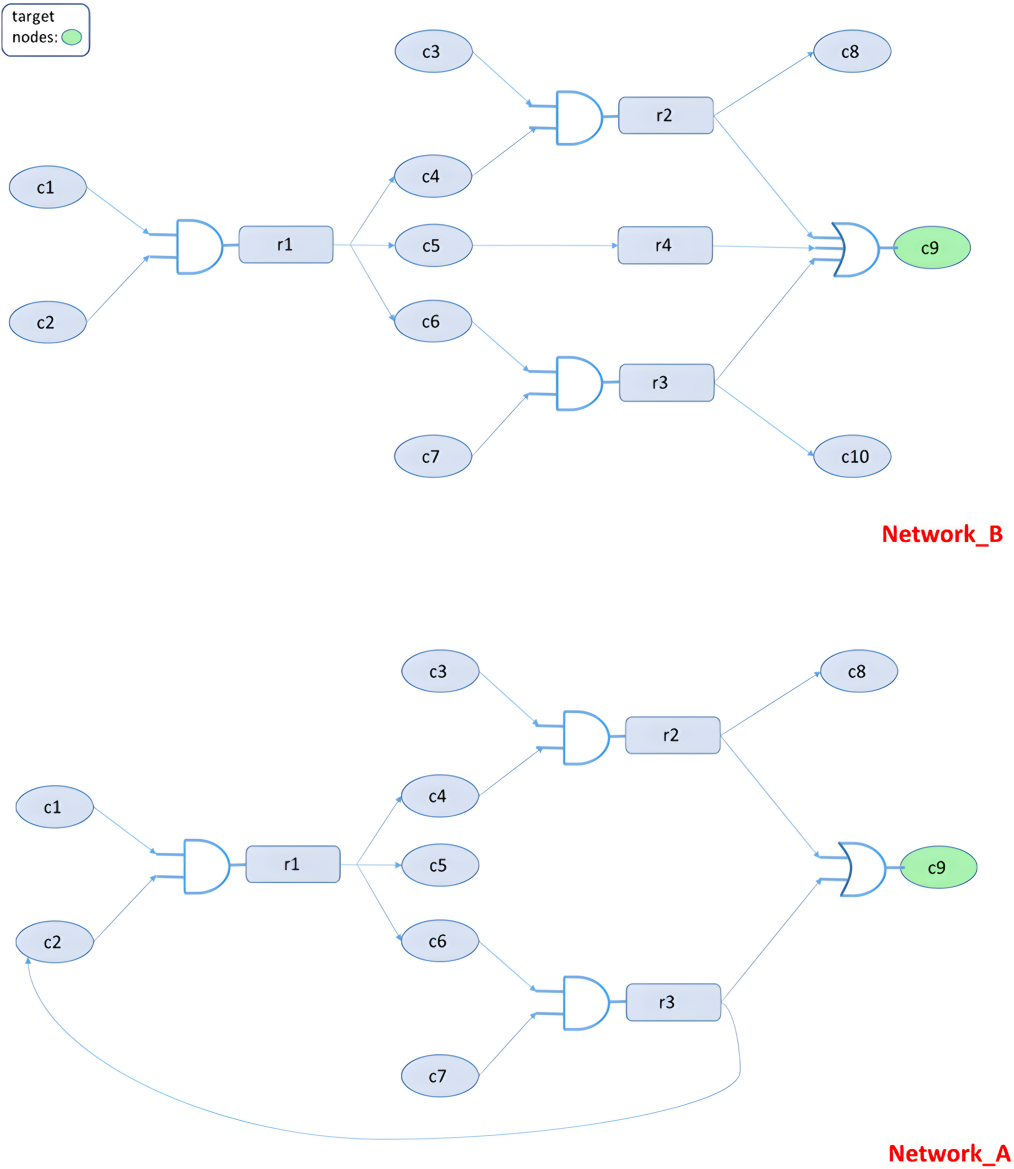
Decomposing the MKMN networks (Lu et al., 2015b) into elementary modes (EMs). Network B includes the edge $c5 \to c9$ (absent in A), while Network A includes the edge $r3 \to c2$ (absent in B). Because of these two differing links, Network B decomposes into three EMs whereas Network A yields a single EM.

We next examine scenarios involving two structurally similar networks, A and B, that differ by two specific edges: the presence of $c5 \to c9$ and the absence of $r3 \to c2$). Our goal is to inhibit a target node C9 in network A while preserving its activity in network B (Fig. 3). Due to a small architectural variation (an additional edge in A), the same perturbation (*e.g*., inhibiting nodes C6, C7, or r3) yields different outcomes across networks. Our model identifies this difference and returns a solution that meets the selectivity criteria. Elementary mode analysis confirms the ILP-derived results.

More specifically, the target node in both networks is C9 (Fig. 3). There is 1 OR and 3 AND gates in network B. C9 activation can be achieved through activating nodes C1-C2, C3-C4, or C6-C7. We can observe the wiring similarity of the two networks, the only difference being the additional r3-C2 linkage in network A. As previously described, the node pairs C1-C2, C3-C4, and C6-C7 can activate target node C9. Since our objective is C9 repression only in network A, the only feasible solutions are the inhibition of C6, C7, or r3 in both circuits. The end result in network A is the inhibition of C2, which in turn inhibits C4, C5 and C6. Thus, the AND gate cannot be activated, and target node C9 will remain inactive. On the other hand, since there is no feedback loop included in network B that could inhibit C2, C9 can still be activated through routes C1-C2-r1-C5-r4-C9 or C3-C4-r2-C8-C9.

By solving the two networks toward unilateral modulation of node *r1* or *r3*, we observe that the solution for node *r3* in Network A follows the route: C1–C2–C4–C5–C6–C7–C9–r1–r3. The elements of this route are a subset of a solution in Network B, which includes the extended route: C1–C2–C4–C5–C6–C7–C9–C10–r1–r3–r4.

**Figure 4:**
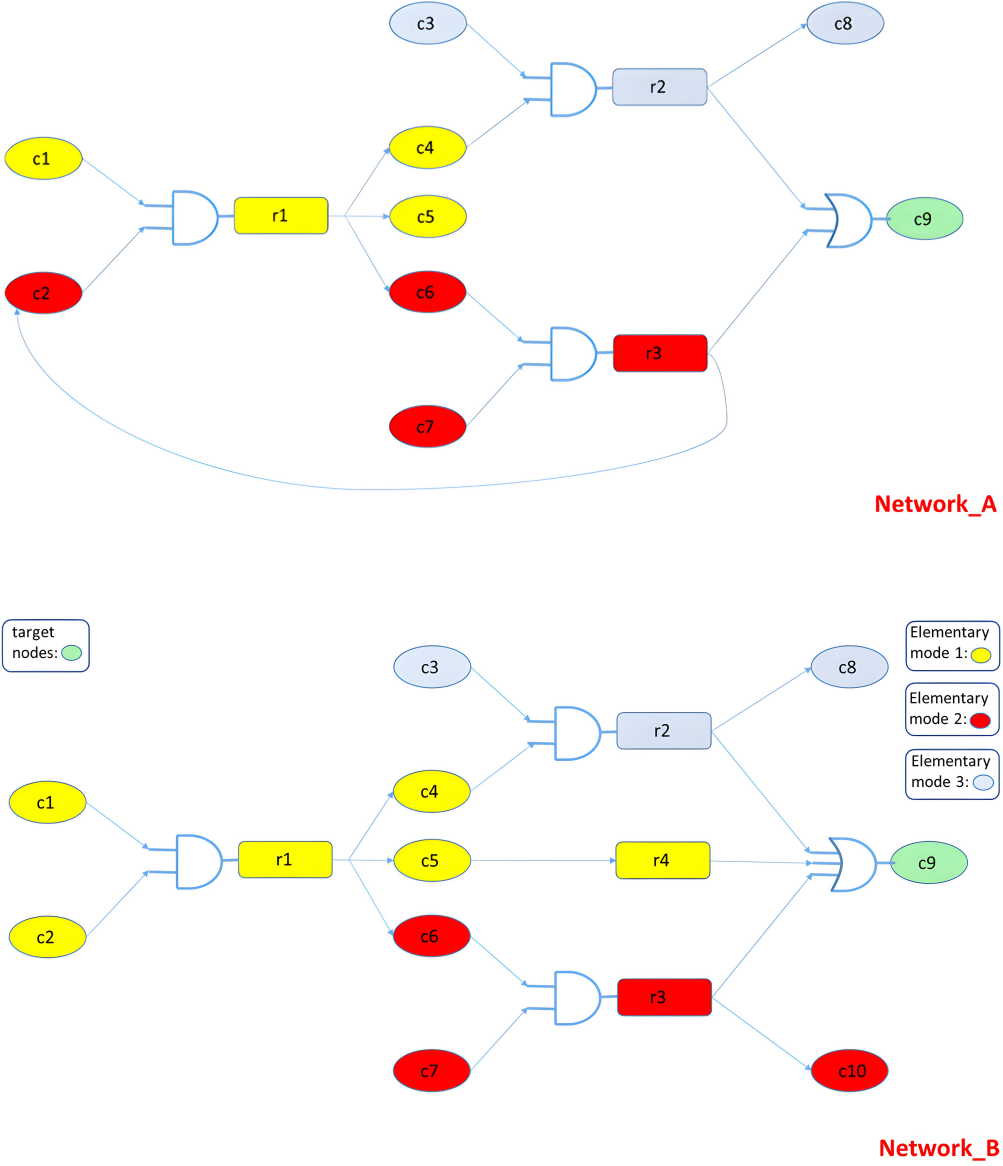
Decomposing the MKMN networks into EMs. There are three EMs in network B, but only one EM in network A.

The first solution involves one elementary mode (EM) in network A: C6–C7–r3–C9, whereas the second involves two EMs in network B: C6–C7–r3–C9 and C1–C2–r1–C5–r4–C9 (Fig. 4). Thus, perturbation of node *r3* in both networks leads to inhibition of all red nodes. However, since the solution in Network A is a subset of that in Network B, the target node remains activated in B through the yellow nodes.

**Figure 5:**
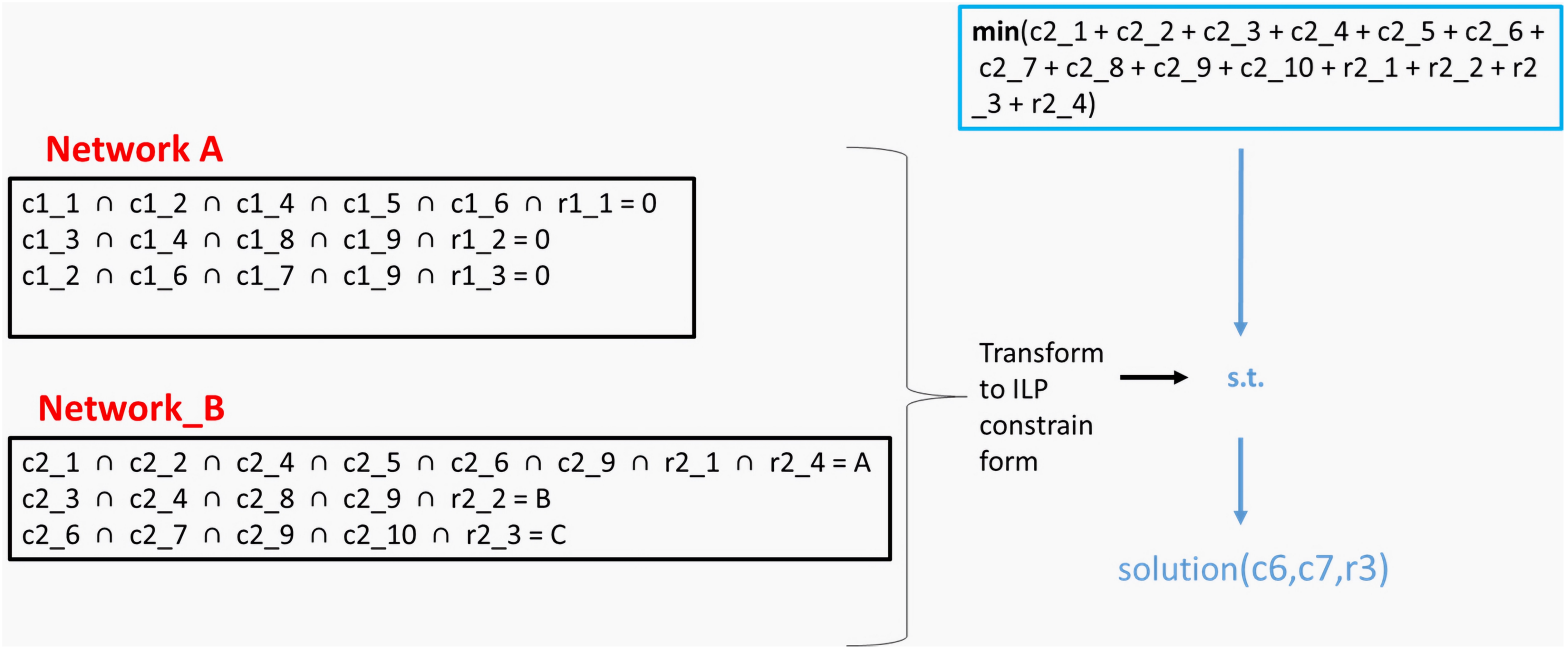
Converting MKMN EMs into linear constraints.

To confirm our results, we solve the same problem by transforming the EMs of each network to integer linear programming constraints, setting the A constraints equal to zero and the B constraints equal to one, and minimizing the sum of the node values of both networks. The returned solution confirms our results by suggesting nodes C6, C7 and r3 (Fig. 5).

**Figure 6:**
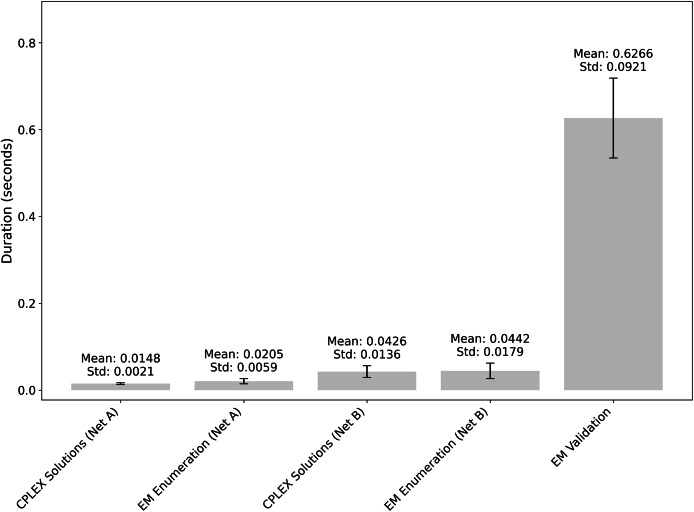
Comparison of runtimes for the ILP (CPLEX) and EM-enumeration methods over 100 iterations.

To compare the runtimes between ILP formulation and EM enumeration methods we followed the three-step workflow as summarized in Algorithm 1: first, we generated all optimal ILP solutions for each network; second, we sorted these solutions by objective value and scanned the network A list to identify the first solution that was a subset of any network B solution; and third, we verified whether this candidate qualified as an elementary mode (EM), proceeding to the next higher-objective solution if it did not. In parallel, as a second method for comparison, we exhaustively enumerated all EMs of both networks to validate our subset-selection procedure and to pinpoint the minimal network A EM contained within an EM of network B. Over 100 iterations, the runtimes of the ILP-based and enumeration-based approaches proved comparable, but the EM verification step-confirming that a subset solution satisfies the EM criteria-emerged as the primary bottleneck, taking roughly an order of magnitude longer than either solution generation or EM enumeration (Fig. 6).

### Selective repression of a node included in both networks ( ${{\mathrm{outdegree}}}\; \ne\; 0$)

**Figure 7:**
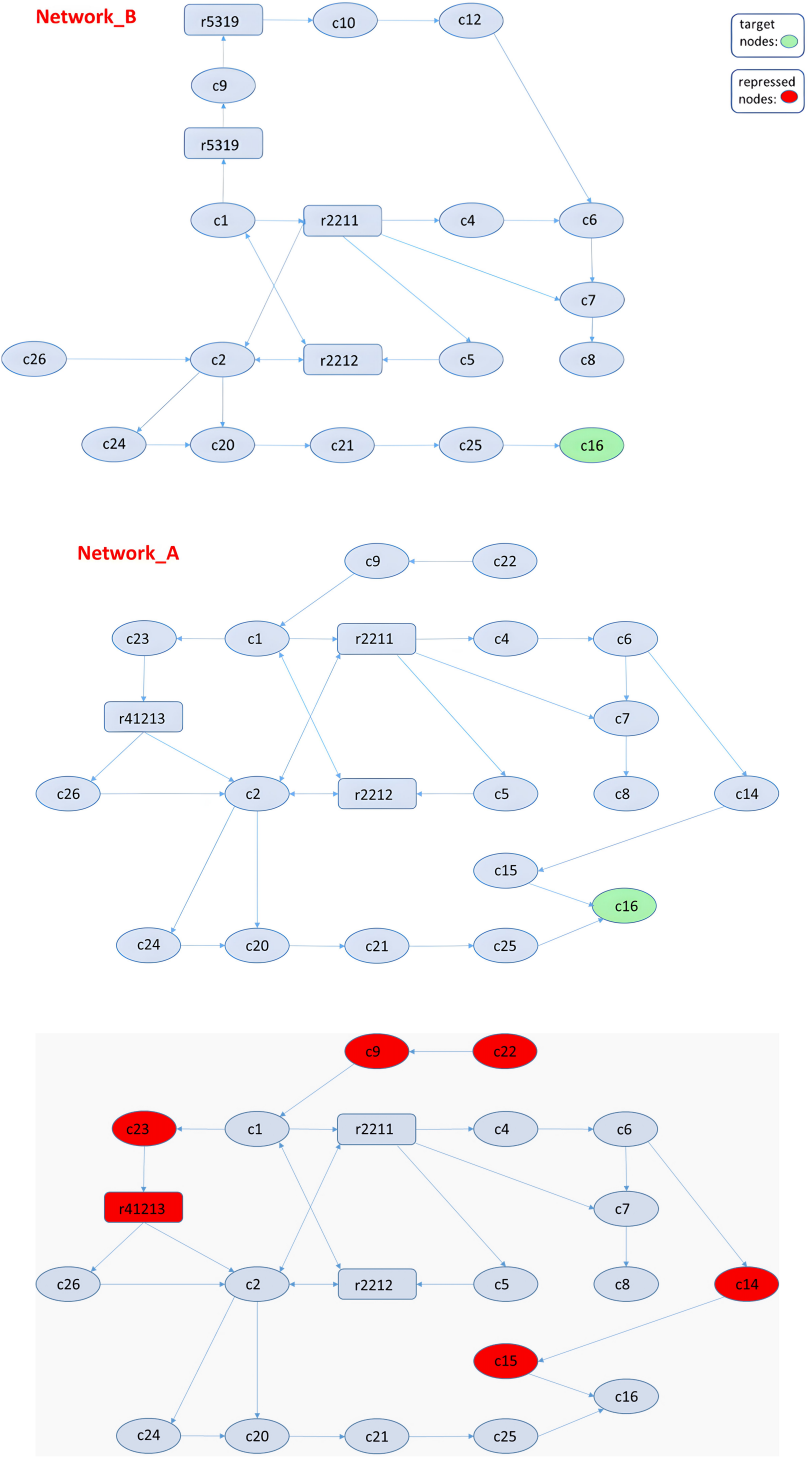
All the returned solutions are displayed color-coded in red. Either repressing C9 in both networks, or C23 or r41213 and C14 or C15 only in network A, the result would be the isolation of C16 in Network B.

In the previous example, the number of nodes in network A was less than that in network B (*N* < *M*). However, in the present example, we analyzed two networks in which *N* > *M*. Our objective is to deactivate node C16 only in network A. In this case, we perturb every node from the total N+M population, and if the returned value is an EM, we select the solution with the minimal objective value. The suggested solutions among common nodes were C9 inhibition and the unique nodes C23 or r41213 and C14 or C15 (based on the objective values, we selected the second nodes in both cases). Thus, to activate node C16 in circuit B and deactivate it in A, our model returned to either inhibiting C9 in both networks or repressing r41213 and C15 in network A (Fig. 7).

### Selective repression of a node uniquely present in one network

**Figure 8:**
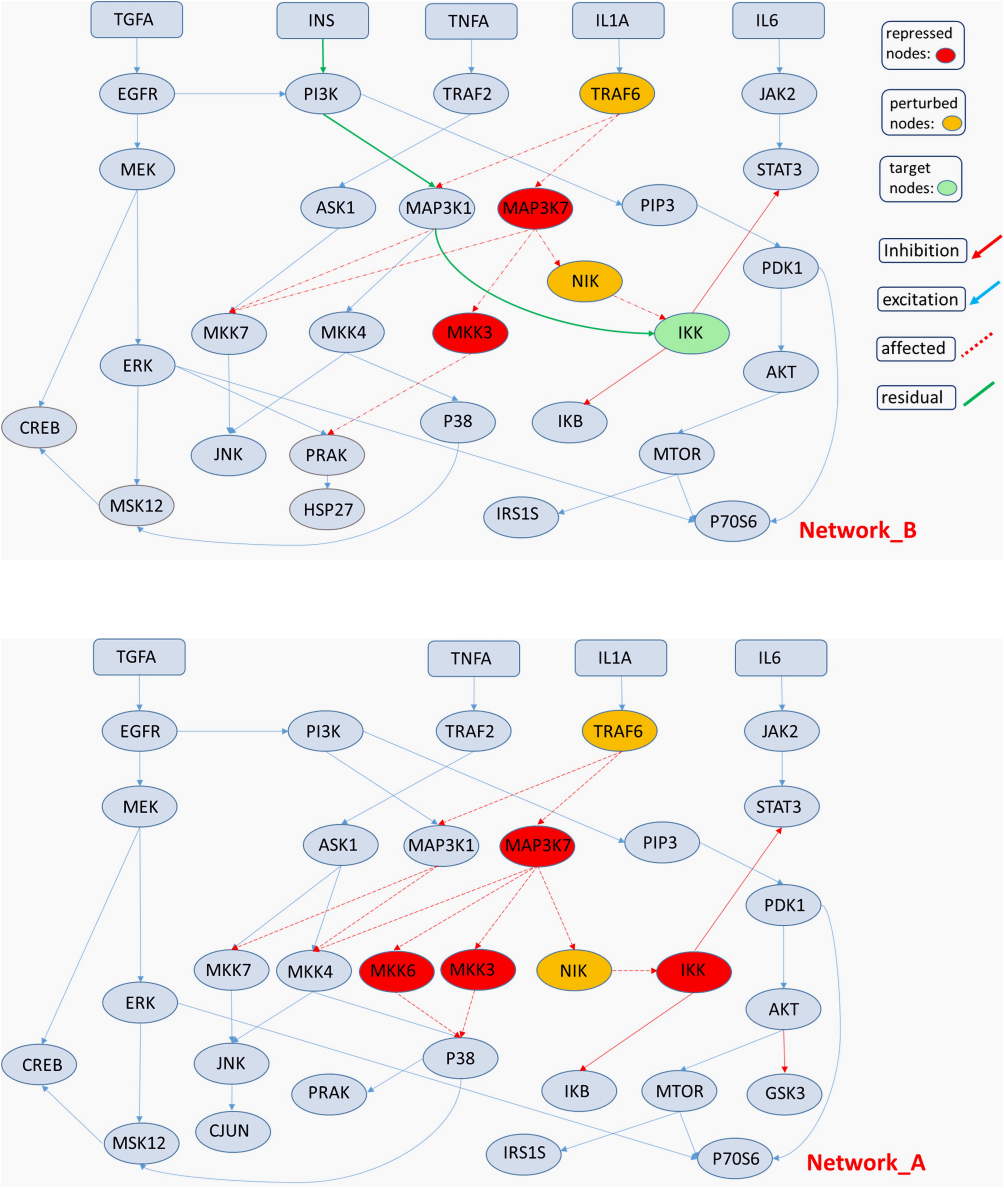
Schematic model illustrating cross-talk between various receptors.

In the final case study, our objective was to deactivate the target node IKK in network A while activating it in network B (Fig. 8). The target node IKK contains both inbound and outbound connections. The main difference between networks A and B lies in a single additional link in network A: a connection between node MAP3K1 and the target node IKK.

In this scenario, there are no central nodes where all connections converge, the target node has outbound links, and we employ Eqs. (2)–(8). As before, we explore the solution space using both EMs and ILP. Our method returned a pool of 5,567 solutions for network A and 4,516 solutions for network B. The solutions obtained by the two methods converge on the NIK–IKK pair (Fig. 8). We observe that the only node preceding IKK is NIK, and its deactivation yields the desired modulation.

In network B, IKK receives inputs not only from NIK but also from MAP3K1. Our method identified an additional solution corresponding to a circuit activated *via* TRAF6 (Fig. 8). This solution is an EM and, according to our imposed conditions, is a subset of a solution from network B that includes both the solution from network A and the EM that arises following PI3K activation.

Based on the objective values, we selected to repress node NIK but not TRAF6. The first solution is straightforward, as it consists of IKK’s direct predecessor, NIK. When NIK is inhibited, IKK in network B becomes completely isolated, whereas IKK in network A remains activatable *via* MAP3K1. The second solution is less obvious, as TRAF6 is not directly connected to the target node.

### KEGG networks

Subsequent case studies test selective intervention under more complex conditions, including varying node counts across networks and different logic gate arrangements. In all cases, the method reliably identifies node sets that drive opposing outcomes in the target node. For validation, we applied the method to real metabolic KEGG networks (CPR1200 and BLJ1200) and showed successful selective repression of the pyruvate node in one network, with its production preserved in the other.

All the included modules are delineated by pink lines in Figs. S1, S2, S3, and S4 in the Supplemental Material. Our objective was to deactivate the node pyruvate in only one of two networks. There were 58 common nodes between the two networks, and each network included four unique nodes. To test our model for slight architecture differences, we modified the initial BLJ network by adding AND/OR gates (Figs. 1 and 3, respectively), while the CPR network was left intact. The two gates were added between nodes 5,10-methylene-THF and serine, and in all cases our efforts focused on deactivating pyruvate only in the BLJ (Figs. 2 and 4). The returned nodes that satisfy the conditions are color coded yellow (Figs. 1–4). The repression of every common yellow node in the two networks results in the deactivation of pyruvate only in BLJ, while its production in CPR continues. In Figs. S5–S8 of the Supplemental Material we observe the results of isolating nodes Glyceraldehyde-3P (Figs. 7 and 8) and Glycine (Figs. 5 and 6). Whereas in the BLJ every route toward pyruvate has been blocked, in CPR at least one route remains accessible (Figs. 5 and 7, yellow lines).

These results support the generality and flexibility of the ILP approach across both synthetic and real-world networks.

## Discussion

### Translational applications and biological motivation

Our ILP framework addresses a common challenge in network medicine: selectively targeting a disease-specific pathway while sparing healthy circuits. This is a crucial step in precision therapy (for example, hitting only cancer-cell signaling circuits, or only the epileptic seizure network) without collateral damage to normal cells or functions. In practice, one can build Boolean models for the “healthy” and “pathological” network variants (*e.g*., from genomics or proteomics data) and then apply our ILP method to find minimal gene or protein perturbations that flip a target off in the disease network but leave it on in the normal network (or vice versa). Similar strategies have proven useful in other settings: for instance, Tercan et al. used single-cell data to build Boolean models of two blood cell states and identified transcription factors whose activation or repression reliably drove one cell type into another (Tercan et al., 2022). In other words, they effectively predicted interventions that rewire one regulatory network without globally disrupting others. Our method extends this idea by automating the search for such discriminating targets across any pair (or set) of related networks. By integrating known drug targets or pathway databases into the models, researchers can directly use these ILP-derived intervention sets to guide experimental design or drug-repurposing campaigns, with confidence that the selected targets exploit network differences unique to the pathological state.

### Tumor heterogeneity networks and targeting

In computational network targeting, genes or proteins are commonly prioritized by drug-relevant and disease-relevant attributes. Targets are often filtered to include only those with known ligands or predicted “pocket” structures, or prioritized by tractability scores. For instance, target–disease databases score each gene by tractability (small-molecule or antibody potential) and clinical precedence, steering interventions toward druggable proteins (McDonagh, 2024). In practice, models may impose constraints to select only pre-known drug targets (*e.g*. FDA-approved targets) or add weights favoring tractable nodes.

One typically avoids indiscriminately targeting genes essential for normal cell survival. Popescu et al. (2022) illustrates this by focusing on “disease-specific survivability-essential” genes as control targets and preferring those that are FDA-approved drug targets. In optimization models, essentiality can be implemented as an additional constraint or objective (*e.g*., maximize inclusion of known disease-essential genes, minimize targets essential in normal tissues).

In cancer network models one might restrict interventions to disease-specific “survival-essential” genes and known drug targets, and then minimize the number of such targets, as in Kobayashi’s multi-drug control models (Kobayashi & Hiraishi, 2013). In practice, ILP-style methods have helped identify combination targets in contexts like melanoma (the WNT5A example), and analogous approaches have been discussed for other systems (*e.g*., metabolic engineering or immune signaling) where one seeks a minimal intervention set to drive the network to a healthy state. These concrete studies show that ILP-based optimization can be used to compute selective intervention strategies in Boolean models of real pathways.

Often targets are chosen by network topology (*e.g*., hubs, bottlenecks) or by belonging to key pathways. Some methods incorporate network centrality scores or require that chosen targets cover the relevant signaling module. In multi-objective ILP formulations, such factors might appear as additional terms in the objective (favoring central/highly connected nodes) or as forbidden nodes (excluding high-risk essential hubs).

In summary, biological filters like druggability, expression level, mutation frequency, and essentiality are commonly used to rank or constrain intervention candidates. These criteria can be built into the model as hard constraints (limiting the search to a subset of nodes) or as soft objectives/weights in the optimization. For example, OpenTargets integrates differential expression and tractability into a scored evidence framework (McDonagh, 2024), which is analogous to how Boolean-network intervention models might prioritize one intervention set over another by such biologically relevant scores.

### Proposed experimental benchmarking in cancer

Bulk (or “mixed”) readout denotes a single measurement from a tumour sample (*e.g*., bulk qPCR, RNA-seq or proteomics) that aggregates signals from all coexisting cell subpopulations and can therefore mask clone-specific behavior. To test our targeted-subnetwork strategy experimentally, one can apply the ILP/solution-pool workflow: (1) identify minimal, selective intervention sets that (*in silico*) flip the target node in some network instances while leaving it unchanged in others; (2) simulate the corresponding bulk readouts by aggregating the network outputs for each candidate intervention; (3) run *post-hoc* plausibility checks (*e.g*., EM validation, biological filters) to prioritize interventions for wet-lab validation; and (4) compare predicted *versus* observed changes using effect sizes and simple statistical tests. This predict-and-test loop provides a concrete quantitative assessment of whether perturbing the selected subnetworks isolates pathogenic clones and produces the expected change in the mixed readout, while preserving non-prioritized subnetworks (cf. Mohanty, Datta & Venkatraj (2014)).

### Neural microcircuit dynamics in epilepsy

Epileptic networks involve canonical microcircuits (*e.g*., pyramidal–interneuron loops and feed-forward inhibition) that operate across different dynamic states. In the normal state excitation and inhibition are balanced, whereas in the interictal state focal hyperexcitability produces isolated “microseizures” or spikes—small ensembles of coactive neurons that are quickly contained by inhibitory feedback. During the pre-ictal phase local ensembles become increasingly synchronized along stereotyped trajectories, while nearby circuits often show heightened interneuron (parvalbumin-positive) firing and suppressed principal-cell output. In the ictal state runaway excitation takes over: pyramidal cells burst almost simultaneously, interneuron control collapses, and hypersynchronous waves propagate through the network. Thus, the same underlying circuitry is involved in all four states, but critical cellular or synaptic modifications can switch the balance between healthy and pathological modes (Stöber et al., 2023).

Evidence from recent epilepsy models and experiments indicates that even generalized seizures originate in focal microcircuits and involve common motifs (like feed-forward inhibition). Studies using *in vivo* calcium imaging and electrophysiology show seizures begin as locally synchronized ensembles (microseizures) that then spread as traveling waves. Modeling and recordings imply that transitions to the ictal state involve increased excitatory coupling and weakened inhibitory feedback (altered E/I balance). These findings suggest that epilepsy reflects the same microcircuits operating under different regimes, distinguished by subtle shifts in excitation–inhibition dynamics (Zhao et al., 2024).

The microcircuit description maps directly onto our described framework: treat each canonical element (*i.e*., pyramidal cell populations, distinct interneuron classes, feed-forward/inhibitory motifs) as nodes or small subcircuits in the Boolean networks for the “normal”, “interictal”, “pre-ictal” and “ictal” regimes (networks A/B or network instances). Subtle cellular/synaptic changes that shift E/I balance such as weakened inhibition, increased recurrent excitation or altered coupling, are encoded as small wiring or logic differences between the network instances, and the ILP then searches for minimal perturbation flags ${u_{i}}$ (and corresponding enforced states ${z_{i}}$) that force the modeled target (*i.e*., the pre-ictal to ictal transition) in one network while leaving the normal network unchanged.

Practically, this yields candidate interventions that correspond to up- or down-regulating specific interneuron or pyramidal nodes (or their inputs) to restore the healthy attractor. In practice the ILP “minimal perturbations” map directly onto experimentally testable manipulations by modulating a small set of nodes/inputs whose up- or down-regulation (*e.g*., stimulating or inhibiting specific principal cells or interneurons) has been shown to abort or prevent seizures in closed-loop experiments. Those candidate interventions correspond to concrete modalities used in the literature such as optogenetic and chemogenetic control, electrical stimulation, or even interneuron transplantation, which all act by shifting the local excitation/inhibition balance back toward the healthy attractor the model identifies.

### Synchronous *vs*. asynchronous Boolean dynamics

A core result from Boolean network theory is that point attractors (fixed-point states) are preserved under asynchronous update. Intuitively, if a state satisfies all update rules (so no node would change its value), then it remains stable whether nodes update in lockstep or one-at-a-time. Recent analysis confirms this: Bensussen et al. (2025) prove that asynchronous dynamics conserve fixed-point attractors. In other words, any synchronous steady state remains a steady state under any asynchronous scheme (and *vice versa*). In summary, the fixed-point predictions of ILP or synchronous Boolean models remain valid under asynchronous semantics, whereas periodic or cyclic attractors usually do not persist. This means interventions designed to reach a steady state will usually have the same endpoint under either update scheme. The basins of attraction, however, can differ in extent.

### Computational complexities

The computational tractability of network-level pathway enumeration depends critically on the chosen algorithm. In one paradigm, mixed-integer linear programming (MILP) as implemented in solvers such as CPLEX tackles the NP-hard task of selecting binary on/off variables *via* branch-and-cut algorithms that integrate linear-programming relaxations, cutting planes, and heuristic heuristics (Bertsimas & Tsitsiklis, 1997; Lodi, 2010). Although the worst-case complexity of MILP remains exponential in the number of binaries, empirical studies on sparse, real-world networks demonstrate markedly sub-exponential scaling (*e.g*., quasi-polynomial or mild exponential) as the problem size increases (Abdelmaguid, 2018): doubling the number of binary variables often increases solve time by only a small constant factor (Lodi, 2010; Achterberg, 2009), enabling enumeration of feasible modes in models with dozens or even hundreds of reactions.

By contrast, EM enumeration directly computes all minimal steady-state pathways—equivalent to the extreme rays of the flux cone—and is intrinsically a #P-hard counting problem (Klamt & Stelling, 2003). Moreover, the number of EMs itself can grow exponentially (or faster) with reaction count *R*; even after network compression, genome-scale reconstructions with $R\sim100$ often yield ${10^{6}}$– ${10^{8}}$ modes (Schuster, Fell & Dandekar, 2000; Terzer & Stelling, 2008). Consequently, both brute-force and sophisticated EM algorithms face an inescapable combinatorial explosion, restricting their practical use to only the smallest or most highly reduced networks.

Together, these findings explain why MILP-based solution-pool enumeration with *post hoc* minimality filtering frequently outperforms classical EM enumeration in practice: although both share exponential worst-case bounds, modern MILP solvers’ advanced branching and relaxation techniques dramatically improve runtime growth, whereas EM enumeration remains fundamentally limited by the sheer number of minimal pathways that must be generated.

As a final step, we incorporate a targeted minimality verification on each Network A mode that is a subset of a Network B solution. Specifically, for each candidate mode, we fix the corresponding binary variables and then iteratively deactivate each active reaction, re-solving the MILP for each perturbation to ensure that no proper subset remains feasible; any feasible sub-solve immediately disqualifies the candidate as non-minimal. While MILP solving is NP-hard in theory (Bertsimas & Tsitsiklis, 1997), these sub-problems are substantially smaller—typically involving only the 5 to 10 reactions active in the candidate support, even in genome-scale models (Terzer & Stelling, 2008)—and benefit from solver features such as warm starts and presolve reductions (Lodi, 2010). As a result, the total computational cost grows only linearly with the candidate’s support size, introducing a modest overhead compared to the MILP solving time, while remaining far more tractable than classical EM enumeration, whose complexity grows super-exponentially with network size due to the combinatorial structure of the null-space (Schuster, Fell & Dandekar, 2000; Urbanczik & Wagner, 2005).

### Benefits and challenges of integer linear programming

Identifying optimal intervention strategies in biological networks is central to drug discovery, especially when the goal is to selectively modulate disease-specific pathways without affecting healthy cells. Our study introduces a scalable, structure-based approach that identifies minimal intervention sets capable of modulating a target node in one network while leaving it unaffected in structurally similar counterparts.

Unlike traditional methods that rely on detailed kinetic modeling or exhaustive enumeration of pathways (*e.g*., minimal cut sets or elementary modes), our method operates on the topology of Boolean networks alone. This dramatically reduces computational overhead and allows for the analysis of larger, more complex systems without requiring fully curated genome-scale models or detailed stoichiometry.

In pharmaceutical research, the challenge of minimizing both direct and indirect off-target effects is increasingly critical. Our approach addresses this by enabling the discovery of interventions that are effective in one network context but inert in others. This is particularly relevant for targeting subpopulations of pathogenic cells that differ from their healthy counterparts through subtle network rewiring, such as cancer stem cells, epileptic foci, or bacterial strains within a microbiome.

Despite offering a novel and computationally efficient strategy for discriminative intervention in Boolean networks, our work could be enhanced in several key areas. A primary limitation lies in the narrow empirical scope of the case studies, which—though varied in structure—remain relatively small in scale. To support claims of scalability and applicability to genome-scale networks, the framework should be validated on larger, high-dimensional biological models, such as whole-cell regulatory or signaling networks. Furthermore, the decision-making process underlying the selection of perturbed nodes lacks biological prioritization; integrating domain-specific data, such as tissue-specific expression, gene essentiality, or known druggability, could make the interventions more actionable. Additionally, the binary logic abstraction, while computationally appealing, may oversimplify complex regulatory phenomena—suggesting the need for hybrid approaches that incorporate multi-valued or fuzzy logic to better model biological nuance. Finally, the validation of the method remains largely computational; experimental or literature-based confirmation of the predicted intervention sets would substantively elevate the work’s translational relevance.

While our method focuses on steady-state network structures, it does not capture the dynamic behavior of these systems or their transitions between cellular phenotypes. Ideally, network modulation should not only eliminate a pathogenic state but also redirect the system toward a desired attractor basin, such as restoring a wild-type or healthy phenotype. Relevant studies suggest that the topology of a regulatory network alone can offer insight into its dynamic capabilities, including stability, multi-stability, and controllability. This modulation of complex intracellular, cellular and tissue-level behaviors can provide insight into the ways in which these behaviors can be manipulated and redirected to an alternative desired phenotype (for instance, to revert a mutant cell to a wild-type state (Marques-Pita & Rocha, 2013) or a mature cell to a pluripotent state (Wang & Albert, 2011). It has been shown that multi-stationarity can be captured by logical analysis (Thomas & Kaufman, 2001a; Thomas & Kaufman, 2001b) and that network structure-only methods can predict the properties of network dynamics (Strogatz, 2001; Klemm & Bornholdt, 2005; Zãnudo, Yang & Albert, 2017) and estimate the number of driver variables that impact the stability, robustness, and controllability of gene regulation and biochemical signaling pathways (Shmulevich et al., 2003; Li et al., 2004; Gershenson, Kauffman & Shmulevich, 2006).

In summary, our integer linear programming framework offers an efficient, scalable tool for selective network intervention. By combining logical modeling with optimization, we lay the groundwork for rational target discovery in heterogeneous biological systems where precision is paramount.

## Conclusions

In this work, we introduced a novel ILP framework for identifying minimal, selective intervention strategies in structurally related Boolean networks. By translating Boolean logic directly into linear constraints and postponing EM analysis to a verification step, our method circumvents the exponential blow-up of full EM enumeration and remains tractable on both toy models and medium-scale metabolic networks.

Our three-step workflow—(1) generating all optimal ILP solutions for networks A and B, (2) ordering these by objective value and selecting the first network A solution that is a subset of a network B solution, and (3) verifying whether that subset qualifies as an EM—proved effective across five case studies. Over 100 iterations, the mean runtimes of the ILP-based search and the exhaustive EM enumeration method were comparable. Importantly, this ILP framework can be straightforwardly scaled to analyze not just two but dozens—or even hundreds—of related networks in a single batch, making it well-suited for large comparative studies or high-throughput intervention screening.

Crucially, we found that EM verification, despite requiring a polynomial-time check per candidate, was the dominant cost in our experiments—roughly an order of magnitude slower than solution generation or enumeration. However, unlike enumeration (which scales exponentially and becomes infeasible on large systems), the verification step scales only polynomially with network size and number of active reactions. For genome-scale networks, EM enumeration is no longer viable, whereas EM verification—especially when paired with early stopping and solver warm-starts—remains a manageable overhead.

Looking forward, our ILP framework offers a scalable foundation for precision interventions in large Boolean models. Incorporating biological priors (*e.g*., gene essentiality or expression data) and extending to multi-valued or dynamic logics could further enhance translational relevance. Ultimately, experimental validation of predicted intervention sets will be key to realizing network-based strategies for targeted therapy with minimal off-target effects.

Finally, in the current work we formulate the problem around a single designated target index ${x^*}$. All ILP decision variables, constraints, the objective function, and Algorithm 1 are written to manipulate a single target node *via* the target constraints $x_{A}^* = {d_{A}}$ and $x_{B}^* = {d_{B}}$. Extending the framework to multi-target interventions would require introducing per-target constraints or a vectorized target variable together with corresponding changes to the objective and constraint logic to resolve trade-offs between targets; this extension is beyond the scope of the present article and is planned as future work.

### Computational environment and timing protocol

All the experiments were performed in the Google Colab environment. The two pipelines were timed by measuring wall-clock duration for each key step repeated over multiple iterations. Those per-run timings were captured with Python’s time functions time.time() or time.perf_counter(). The selected solver and libraries are IBM ILOG CPLEX Optimizer v22.1.2.0 and DOcplex v2.29.245.

## Supplemental Information

10.7717/peerj.20676/supp-1 Supplemental Information 1 KEGG circuits.

10.7717/peerj.20676/supp-2 Supplemental Information 2 Graph generation and analysis.

## Additional Information and Declarations

### Data Availability

The following information was supplied regarding data availability:

The codes used in the current work for graph generation and analysis are available in the Supplemental File.
