# Supplementary material for: Integer linear programming for contrasting state interventions in Boolean networks
Source: PeerJ. 2026 Mar 6;14:e20676. doi: 10.7717/peerj.20676 (PMC12970315; doi:10.7717/peerj.20676)
Supplement: Supplemental Information 1 [file peerj-14-20676-s001.pdf]

BLJ network

APPENDIX

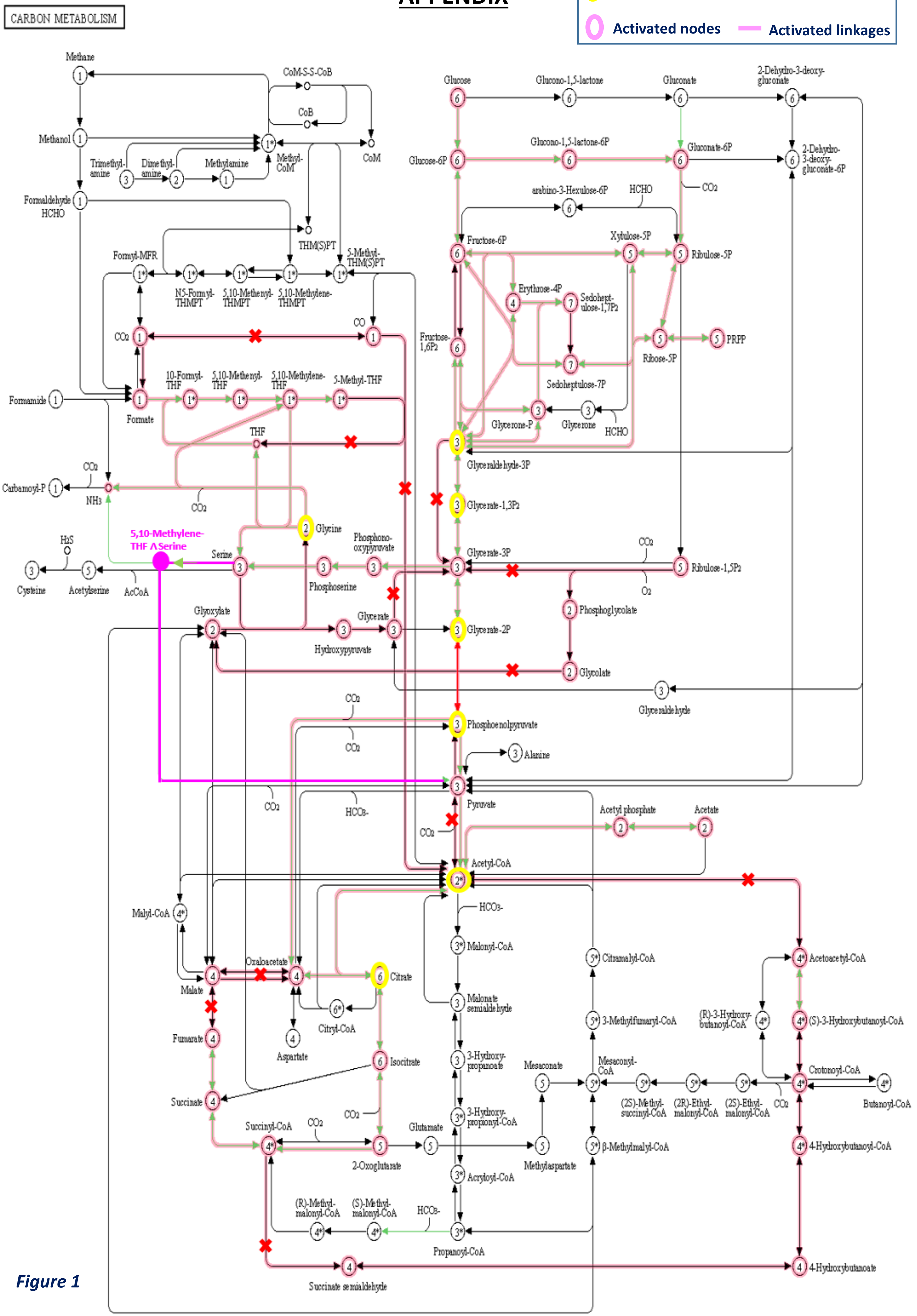

Figure 1

CPR network

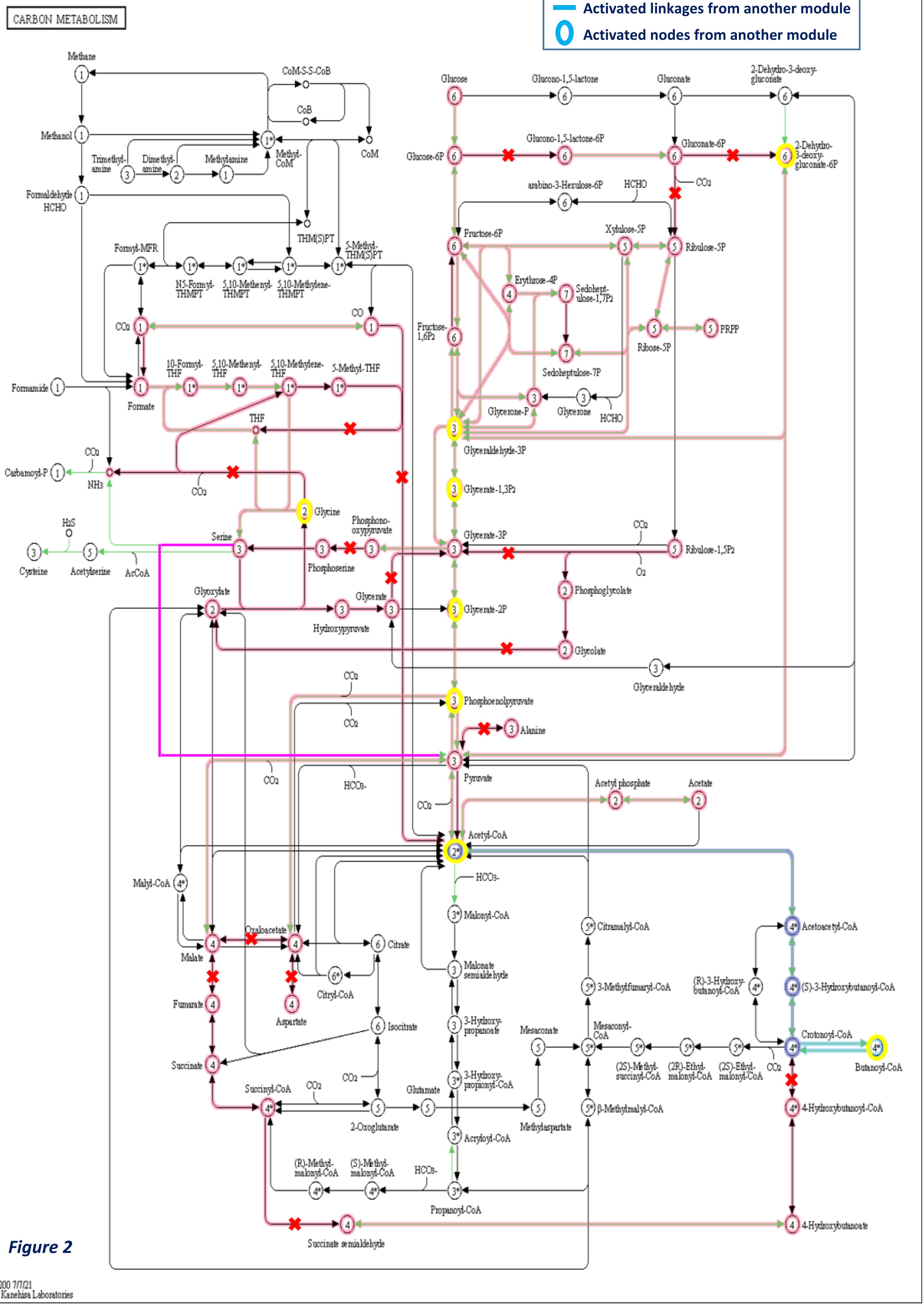

Figure 2

BLJ network

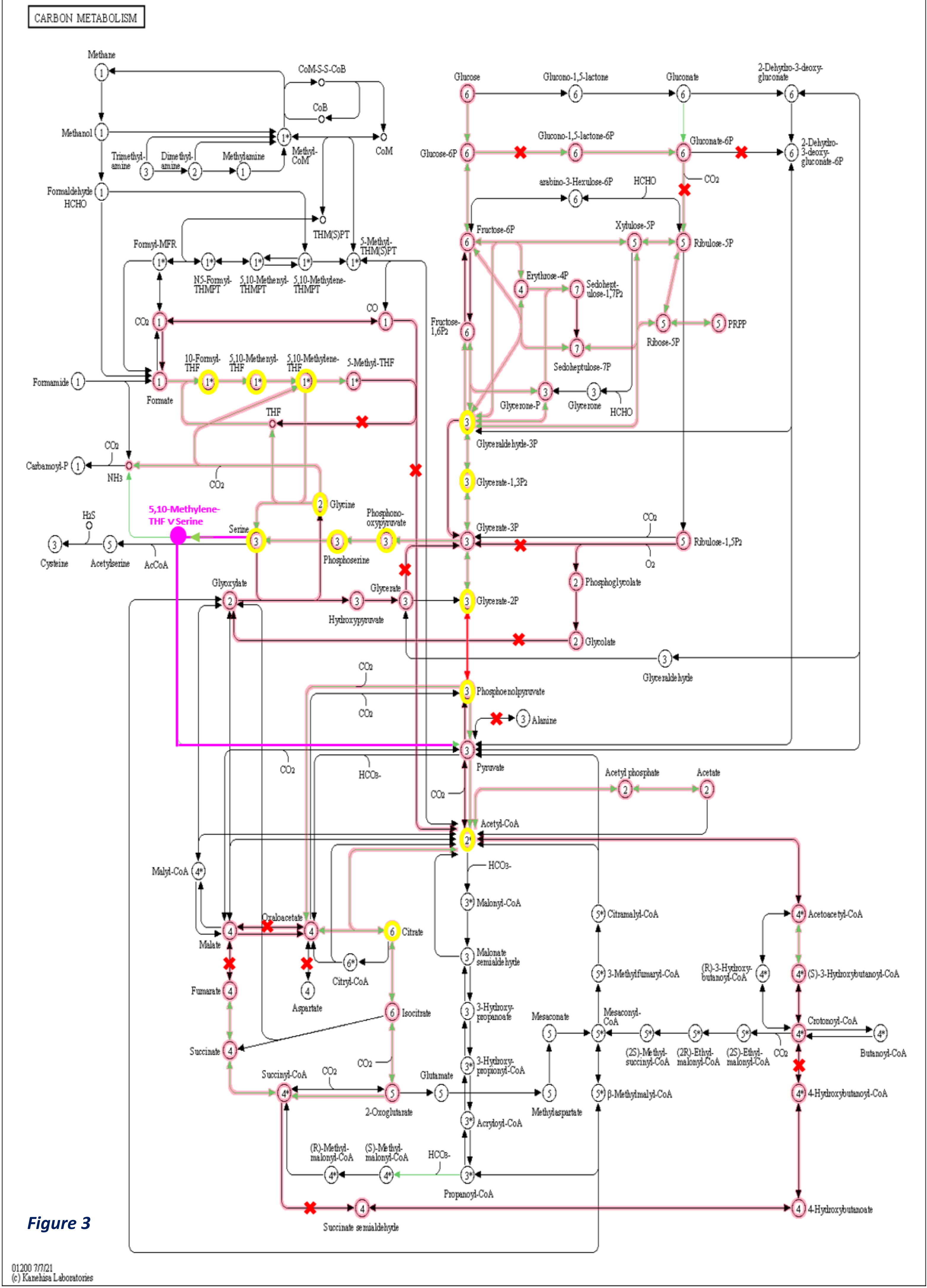

Figure 3

CPR network

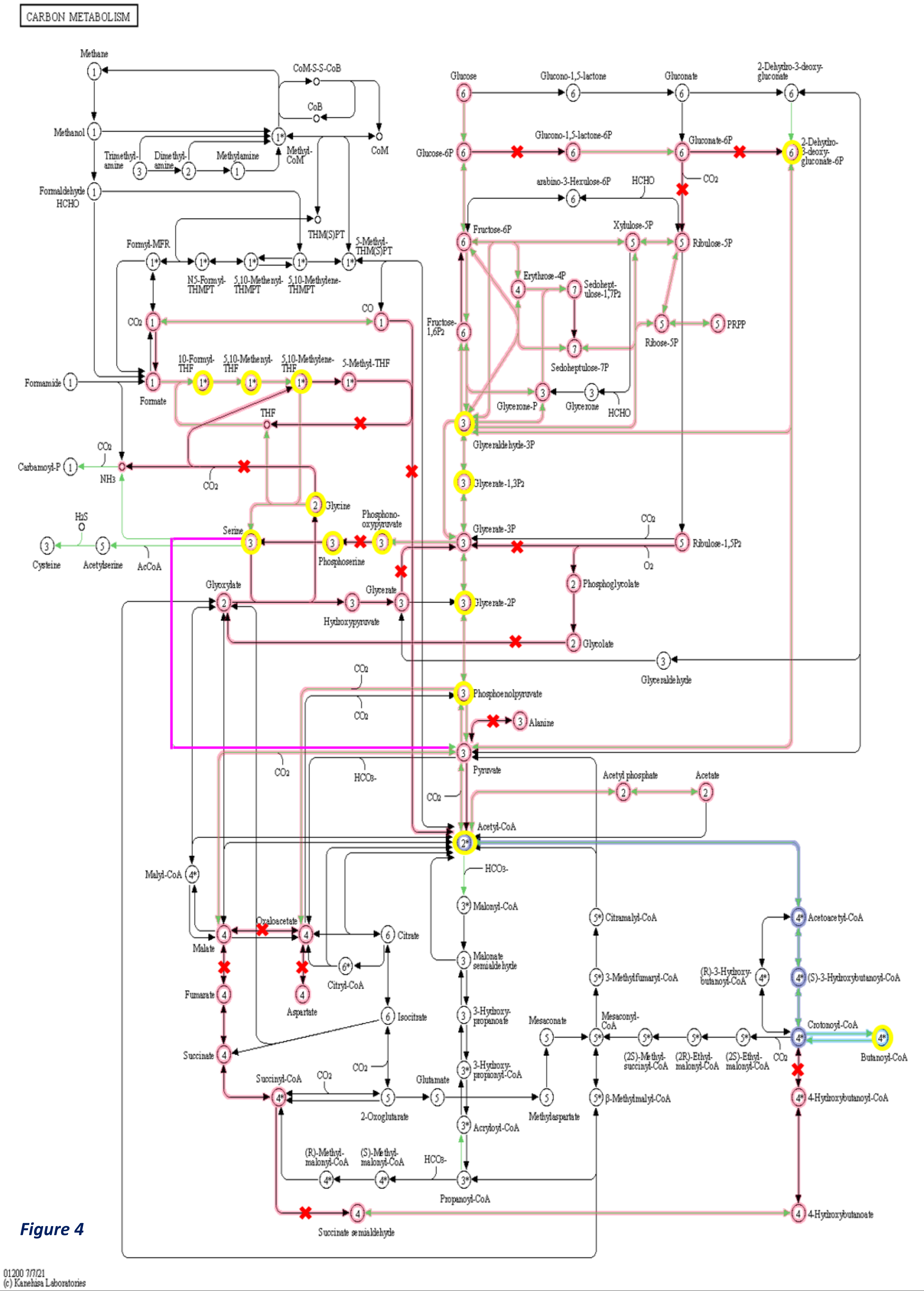

Figure 4

CPR network

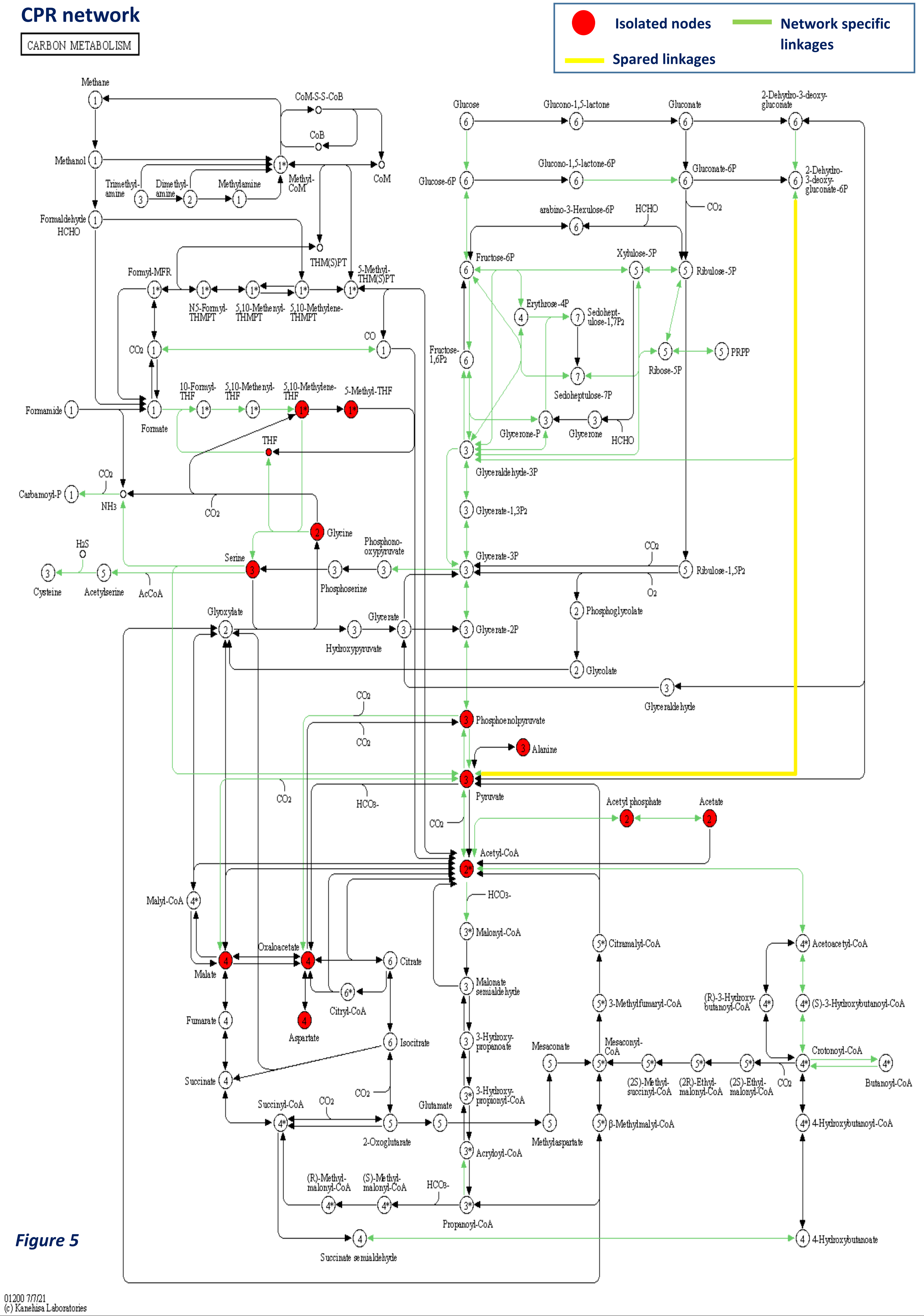

Figure 5

BLJ network

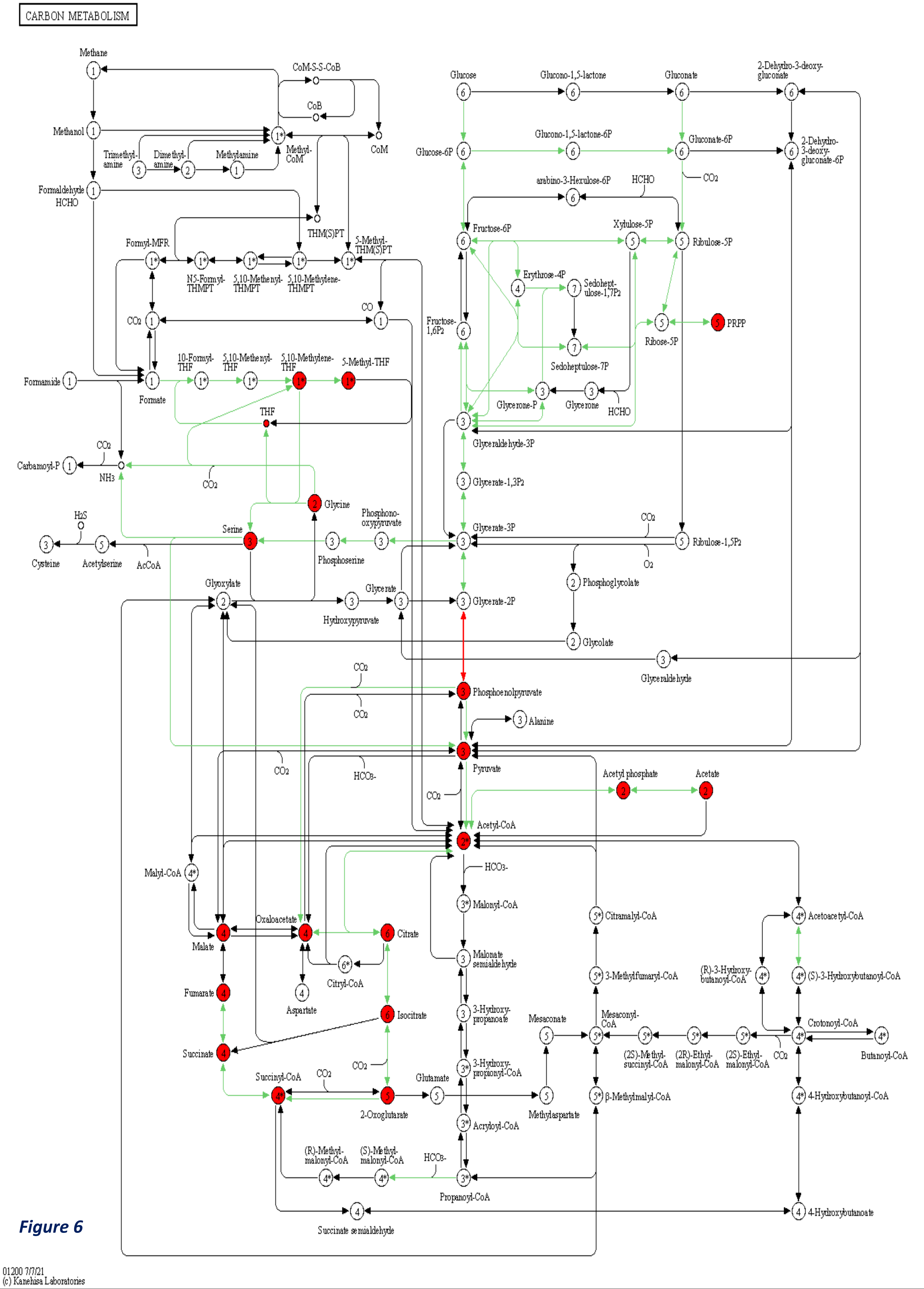

Figure 6

CPR network

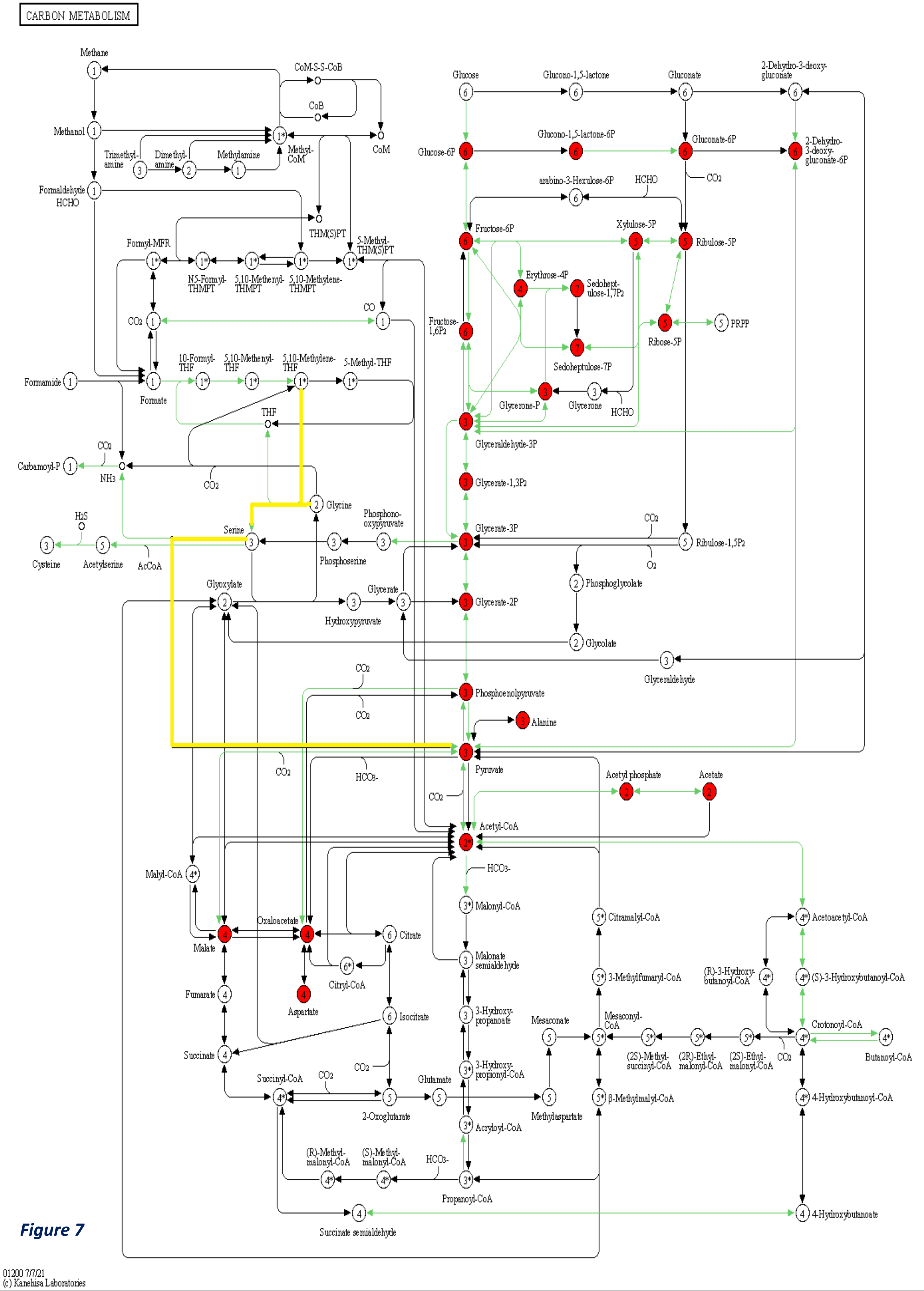

Figure 7

BLJ network

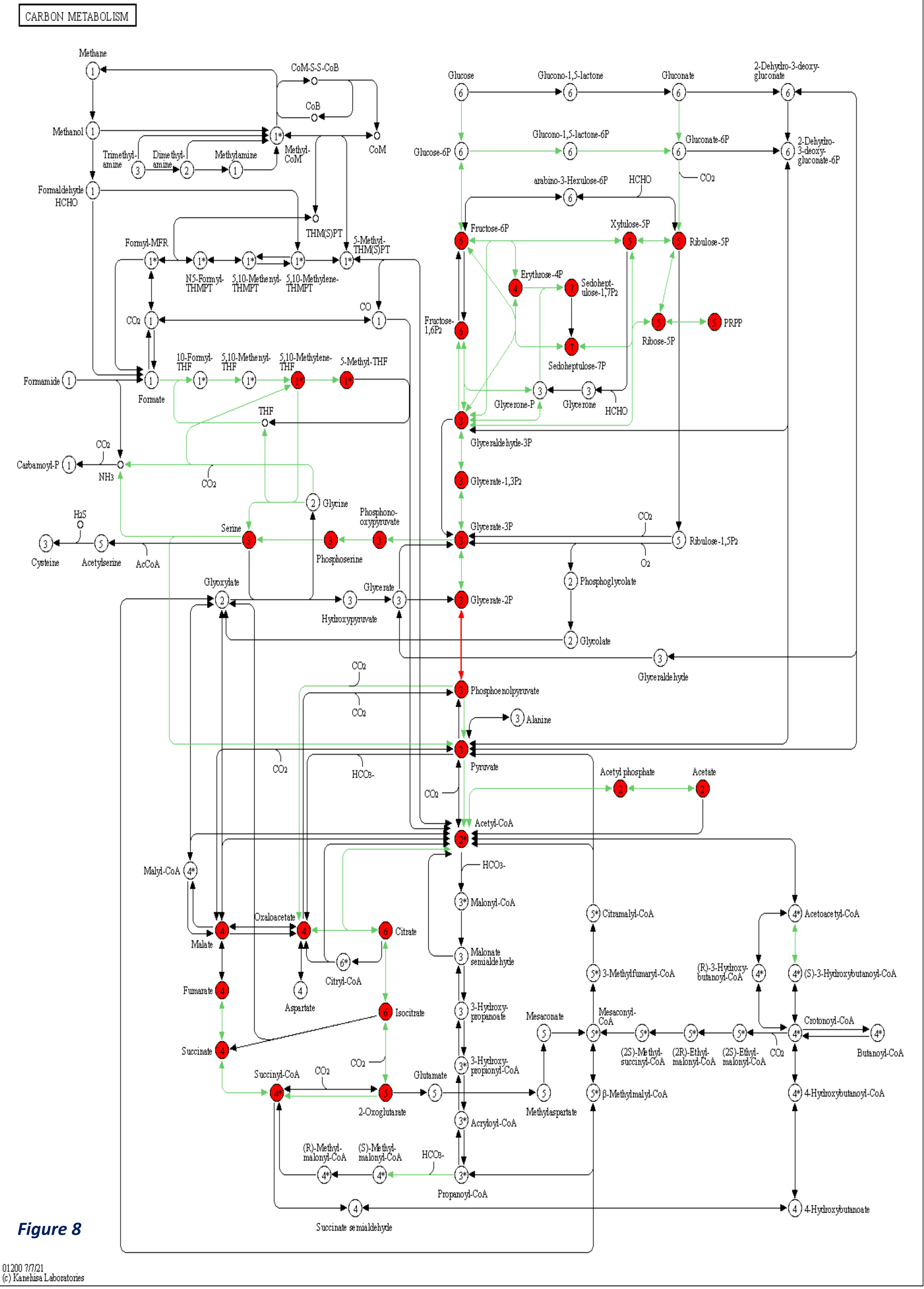

Figure 8
